# T-Follicular-Like CD8^+^ T Cell Responses in Chronic HIV Infection Are Associated With Virus Control and Antibody Isotype Switching to IgG

**DOI:** 10.3389/fimmu.2022.928039

**Published:** 2022-06-15

**Authors:** Luis Romero-Martín, Ferran Tarrés-Freixas, Núria Pedreño-López, Maria L. Rodríguez de la Concepción, Francesc Cunyat, Dennis Hartigan-O'Connor, Jorge Carrillo, Beatriz Mothe, Julià Blanco, Marta Ruiz-Riol, Christian Brander, Alex Olvera

**Affiliations:** ^1^IrsiCaixa AIDS Research Institute, Hospital Germans Trias i Pujol, Institute for Health Science Research Germans Trias i Pujol (IGTP), Badalona, Spain; ^2^Departament de Biologia Cellular, de Fisiologia i d’Immunologia, Universitat Autonoma de Barcelona, Cerdanyola del Valles, Spain; ^3^Department of Medical Microbiology and Immunology, University of California, Davis, Davis, CA, United States; ^4^California National Primate Research Center, University of California, Davis, Davis, CA, United States; ^5^Division of Experimental Medicine, University of California, San Francisco, San Francisco, CA, United States; ^6^CIBERINFEC, Centro de Investigación Biomédica en Red, Instituto de salud Carlos III, Madrid, Spain; ^7^University of Vic-Central University of Catalonia (UVic-UCC), Vic, Spain; ^8^Fundació Lluita contra la Sida, Infectious Disease Department, Hospital Universitari Germans Trias i Pujol, Badalona, Spain; ^9^Catalan Institution for Research and Advanced Studies (ICREA), Barcelona, Spain; ^10^AELIX Therapeutics, Barcelona, Spain

**Keywords:** HIV, humoral immune response, human immunodeficiency virus (HIV) control, T-follicular cytotoxic (Tfc) cells, viral control

## Abstract

T cell responses are considered critical for the *in vivo* control of HIV, but the contribution of different T cell subsets to this control remains unclear. Using a boosted flow cytometric approach that is able to differentiate CD4^+^ and CD8^+^ T cell Th1/Tc1, Th2/Tc2, Th17/Tc17, Treg and Tfh/Tfc-like HIV-specific T cell populations, we identified CD8^+^ Tfc responses that were related to HIV plasma viral loads and associated with rate of antibody isotype class switching to IgG. This favorable balance towards IgG responses positively correlated with increased virus neutralization, higher avidity of neutralizing antibodies and more potent antibody-dependent cell cytotoxicity (ADCC) in PBMCs from HIV controllers compared to non-controllers. Our results identified the CD8^+^ Tfc-like T-cell response as a component of effective virus control which could possibly be exploited therapeutically.

## Introduction

A small percentage of HIV-infected individuals (<1%, referred to as *HIV controllers*) naturally control viral replication in the absence of highly-active antiretroviral therapy (HAART), likely by innate and adaptive immune mechanisms (1, 2). Detailed understanding of this natural immune-mediated control is critical for the development of effective immune strategies for the prevention and, especially, the cure of HIV infection (2). Specific HLA class I genotypes have shown associations with viral control, possibly by restricting efficient cytotoxic CD8^+^ T-cell responses (CTL or Tc) (3). However, this effect only accounts for 10-15% of the disease course variability in untreated hosts (4). Moreover, the relative contribution of different CTL specificities to overall virus control remains controversial due to its high variability among HIV controllers (5, 6). Humoral immune correlates of controlled HIV disease have also been explored in addition to cellular immunity. Passive immunization with broadly neutralizing antibodies (bnAb) has shown compelling clinical efficacy (7), especially when using structurally engineered antibodies. These studies have shown that both, neutralizing and non-neutralizing HIV-specific antibodies can trigger different antiviral effector functions, including Fc-mediated effects such as antibody-dependent cell-mediated cytotoxicity (ADCC) (8). However, naturally-induced, neutralizing antibodies seem to have a limited capacity to control established viral replication and bnAbs are only elicited in a minority of infected individuals. Furthermore, their presence is generally associated with increased HIV replication and diversity (9, 10). The mechanisms that lead to the induction and maintenance of humoral responses with non-neutralization effector functions is less well understood and no study has reported a unique humoral immune correlate of viral control. In addition, relative control of HIV infection may depend on several factors, including not only immunological, but also viral features (11, 12), like viral replicative fitness (11) and mutational immune escape (13).

While it is increasingly accepted that effective HIV control requires the elicitation of both, strong cellular and humoral adaptive responses, the nature of the most appropriate T cell population to support antibody production remains elusive. Thereby, it may be a question of specificity as well as of the polarization of the effector profiles of such T-helper cells (Th). Pathogen-specific memory T-cells can be found in different Th subsets that can have different, or even opposite effector functions (14). While much is known about CD4^+^ T-cells and their differentiation into different Th subsets (such as Th1, Th2 or Th17-like) (14), such classification is less evolved for CD8^+^ T-cells. Interestingly, CD8^+^ T-cells have been recently reported to also differentiate into distinctly polarized subsets, characterized by effector functions other than their classical cytotoxic capacity (Tc) and have been implicated in different mechanisms of immune control (15). For instance, CD8^+^ subtypes with Th2-like phenotype have been associated with effective immune responses to *Mycobacterium tuberculosis* (Mtb), mediated by antigen recognition through non-classical HLA-E (16).

Based on the broad spectrum of effector function (mostly defined by cytokine production signatures), T-cells are thought to acquire three major polarization profiles: type 1 (CD4^+^ Th1 and CD8^+^ Tc1, characterized by the secretion of IFN-γ, IL-2 and TNF-α), type 2 (CD4^+^ Th2 and CD8^+^ Tc2, which mainly produce IL-4, IL-5, IL-10 and IL-13) and type 3 (CD4^+^ Th17 and CD8^+^ Tc17, with IL-17 and IL-22 as signature cytokines) (17). T-cell subset diversity is completed by regulatory T-cells (Treg, either CD4^+^ or CD8^+^, which produce IL-10 and TGF-β) (18), as well as several less characterized, rarer subsets (Th22, Th9 and others) and, importantly, follicular T-cells. Follicular T-helper cells (Tfh) have been shown to play an important role in the development of humoral immunity. Located at the edge of the T-cell zone in secondary lymphoid organs, activated naïve CD4^+^ pre-Tfh cells start expressing CXCR5, PD-1 and ICOS (19) and migrate to the germinal center of the follicles. There, they interact with B-cells, supporting their proliferation, isotype class switching and antibody affinity maturation through cyclic somatic hypermutations. Tfh functionality is related to IL-4 and IL-21 secretion, which is necessary for the establishment of antigen-specific, long-lived plasma cells and circulating memory B-cells (20). Besides, CD4^+^ Tfh cells have been reported to promote the generation of high-avidity antibodies by driving B cells through somatic hypermutations (21). Although it was initially thought that follicle-organizing properties are reserved to CD4^+^ T-cells, there is growing evidence that CXCR5-expressing CD8^+^ T-cells also localize in, or proximal to, B-cell follicles. These CD8^+^ T follicular cytotoxic cells (Tfc) have been reported to have a self-renewal and less-exhausted profile and to keep their cytotoxic functionality to eliminate infected cells while also contributing to the regulation of the antibody response (22).

The association between type 1 responses and reduced HIV viremia has been demonstrated for CD8^+^ (23) and CD4^+^ (24) T-cells. Although postulated years ago (25), the importance of other T-cell subsets exerting alternative effector functions (not mediated by IFN-γ) in HIV pathogenesis remains less well studied. This gap might be partly explained by factors including the small size of some subsets in peripheral blood, their heterogeneity and plasticity, or the severe CD4^+^ T-cell depletion suffered during acute HIV infection (26). In the early 1990’s, the CD4^+^ Th1/Th2 balance was considered a hallmark of HIV disease progression, although that view was not uniformly shared (27, 28). Nowadays, while HIV-specific type 2 and type 3 responses remain poorly understood, the relevance of CD4^+^ Th2 and Th17 in disease control and vaccine response begins to emerge (25, 29–33). Conversely, HIV-specific CD8^+^ Tc2 responses are associated with reduced cytotoxic activity (34), while the preservation of tissue-resident Tc17 during HIV infection is important to regulate immune activation (35). Regulatory CD4^+^ T-cells have been associated with weaker HIV-specific cellular immune responses and been shown to suppress HIV-induced immune hyperactivation and thus, limit infection of conventional CD4^+^ T-cells (29, 30). CD4^+^ Tfh cells have been reported to have different HIV antigen specificities compared to conventional CD4^+^ Th1 cells and to provide help to differentiate and mature HIV-specific B-cells, while supporting CTL responses (36–38). In comparison, Tfh cells seem to be very permissive to HIV entry and contain a significant proportion of the latent viral reservoir (39, 40) and expansion of this cell subset is associated with disease progression (41, 42). Less is known about CD8^+^ Tfc cells in HIV infection, with some studies reporting a higher *ex vivo* cytotoxic activity compared to non-CD8^+^ Tfc (43). These findings are in line with the possibility that CD8^+^ T-cells having Tfc and Tc2-like phenotypes mediate lower viral loads in animal models of chronic infection, including lymphocytic choriomeningitis virus (LCMV)-infected mice (44) and SIV-infected macaques (45). These observations support the hypothesis that CD8^+^ Tfc T-cells, with increased cytotoxic capacity and localized to lymph node follicles, are critical in limiting the viral reservoir established in CD4^+^ Tfh T-cells and provide additional help for maturation of humoral immunity in the follicles (46).

Since such unconventional profiles might be easily missed by standard methodologies and low expression of some of the cytokines, here we applied a novel flow cytometric approach referred to as *Boosted Flow*, which was designed to identify the broadest T-cell polarization profiles of antigen-specific T-cells in a specific and sensitive manner (47, 48). We applied *Boosted Flow* analyses to screen cellular immunity against the full HIV proteome in HIV-infected individuals with variable ability to control their infection to identify specific polarized responses that might be related to HIV control.

## Material and Methods

### Study Design

The aim of this study was to identify HIV-specific T-cell responses with alternative (non-IFN-γ- mediated) effector function profiles that are associated with critical aspects of the humoral immune responses to HIV. Forty-four HIV-infected, treatment-naïve individuals (HIV^+^) and 8 HIV-seronegative controls (HIV^-^) were included in this study. Twenty-four participants had plasma viral loads (pVL) <2000 copies/mL and were considered HIV controllers (C). An additional 20 participants presenting pVL >5000 copies/mL were considered non-controllers (NC). Plasma samples from all the individuals were tested for anti-Env IgM, IgA and IgG titers as well as plasma neutralization titers and ADCC activity. Due to sample availability, boosted flow analysis for cellular responses included 29 individuals of them (15 HIV controllers and 14 HIV non-controllers) and phenotypic characterization based on Th2/Tc2 markers (CRTH2) and Tfh/Tfc markers (CXCR5^+^ PD-1^+^ ICOS^+^) was performed in 22 of the samples used in the boosted flow analysis (8 HIV controllers and 14 HIV non-controllers). Clinical parameters and number of samples included in each experiment are summarized in **Table 1**. Additional clinical and demographic data on the study cohort are included in **Supplementary Table 1**. *Ex-vivo* experiments were performed without blinding or randomization. The study was carried out in accordance with the recommendations of the Ethics Committee of the Hospital Universitari Germans Trias i Pujol in Badalona, Spain (PI-18-011). All subjects gave written informed consent in accordance with the Declaration of Helsinki.

**Table 1:** Clinical data (CD4 count and plasma viral load) and number of samples included in each experiment.

Group	HIV Controllers	HIV Non-controllers
Fiebig stage	VI	VI
CD4 count (cells/mm3)	786 (364-1638)	406 (6-986)
Plasma viral load (HIV RNA copies/ml)	210 (25-1900)	52435 (5576 - 640803)
*Boosted flow*	n=15	n=14
IFN-γ, IL-4 and IL-21 ELISpot	n=8	n=14
Polarization markers	n=8	n=14
IgM/IgA/IgG titers	n=24	n=20
Avidity	n=24	n=20
Neutralization assay	n=24	n=20
ADCC	n=24	n=20

### *Boosted Flow* Screening of HIV-Specific T-Cell Responses

Cryopreserved peripheral blood mononuclear cells (PBMC) from 15 HIV^+^ controllers and 14 HIV^+^ non-controllers were thawed and stained. We cultured 5x10^5^ PBMC/well in R10 (RPMI medium (Gibco) supplemented with 2 mM L-glutamine (Gibco), 100U/ml penicillin (Gibco), 100 µg/mL streptomycin (Gibco) and 10% heat-inactivated fetal bovine serum (FBS – Invitrogen), for 16h at 37°C. PBMCs were stimulated with a set of 425 18mer overlapping peptides (OLPs) divided in 17 peptide pools (range 21-27 OLPs/pool, final concentration for each peptide 10 µg/mL) covering all the HIV proteome, in the presence of anti-CD49d and anti-CD28 and GolgiStop (BD). Anti-CD3/anti-CD28 magnetic beads (Gibco) were used as positive control. As negative controls, culture medium was used instead of peptide pools. Cells were stained first with BD Horizon™ Fixable Viability Stain 575V, followed by extracellular staining for T-cell lineage markers (anti-CD3 APC-Cy7, anti-CD4 BV786, anti CD8-PerCP, anti-CCR7 BV711 – Biolegend; anti-CD45RA FITC – BD), B-cell lineage marker (anti-CD19 PE-Dazzle594 – Biolegend) and myeloid lineage marker (anti-CD14 A700 – Biolegend). Following the fixation and permeabilization step (Fix and Perm kit - Invitrogen), intracellular staining (ICS) was performed, following the boosting strategy established by Ruiz-Riol et al. (48) for Type 1, Type 2, Type 3, Treg and T follicular defining cytokines. Antibodies against Type 1 related cytokines were conjugated with BV605 (anti-IFN-γ, anti-IL-2, anti-TNF-α – Biolegend), antibodies against Type 2 related cytokines with BV421 (anti-IL-4, anti-IL-10, anti-IL-13 – Biolegend), antibodies against Type 3 related cytokines with PE-Cy7 (anti-IL-17-A, anti-IL-22 – Biolegend), antibodies against Treg related cytokines with PE (anti-IL-10, anti-LAP – Biolegend) and antibodies against T follicular related cytokines with APC (anti-IL-4, anti-IL-21 – Biolegend). Different clones were used for the detection of IL-4 in type 2 and follicular. Approximately 10^5^ cells were acquired on a LSR Fortessa BD Instrument and analysis was performed using FlowJo 10.5.2. The gating strategy is summarized on **Supplementary Figure S1**. Cells were classified as T_CM_ (CD45RA^-^ CCR7^+^), T_EM_ (CD45RA^-^ CCR7^-^), T_EMRA_ (CD45RA^+^ CCR7^-^) and T_Naive_ (CD45RA^+^ CCR7^+^). The percentage of cytokine producing cells (magnitude) detected in unstimulated controls was subtracted from the magnitude of the antigen-stimulated cells for each individual. The value of the most negative magnitude value control subtraction across all individuals determined the threshold for positive responses, as described by Roederer et al. (49). Polyfunctional cytokine profiling was done applying Boolean gates in FlowJo 10.5.2, following the same strategy for unstimulated cell signal subtraction and represented using SPICE v6 (provided by the National Institute of Health, Mario Roederer, ImmunoTecnhnology Section, Vaccine Research Center, NIAID, NIH, Bethesda).

### Phenotypic Characterization of CD4^+^ Th2- and Tfh Cells and CD8^+^ Tc2- and Tfc- Cells

Surface markers for Th2/Tc2 (CRTH2) and Tfh/Tfc T cell polarization (CXCR5, ICOS and PD-1) were characterized in cryopreserved PBMC from patients previously yielding CD8^+^ Tfc responses in the boosted flow whole proteome analysis. Cells were thawed and plated at 5x10^5^ PBMCs/well in R10 for 16h at 37°C. PBMCs were stimulated with peptide pools that showed activation of CD8^+^ Tfc in the initial screening or control stimulations as above. Cells were stained first with BD Horizon™ Fixable Viability Stain 575V, followed by extracellular staining for T-cell lineage markers (anti-CD3 APC-Cy7, anti-CD4 BV786, anti-CD8-PerCP, anti-CCR7 BV711 – Biolegend; anti-CD45RA FITC – BD), Th2/Tc2 marker (anti-CRTH2 BV605 – Biolegend) and Tfh/Tfc markers (anti-CXCR5 PE, anti-ICOS PE-Dazzle594, anti-PD-1 PE-Cy7 – Biolegend). Following the fixation and permeabilization step (Fix and Perm kit - Invitrogen), intracellular staining was performed using anti-IL-4 BV421 and anti-IL-21 APC (Biolegend).

Approximately 10^5^ cells were acquired on a LSR Fortessa BD Instrument and analysis was performed using FlowJo 10.5.2 software. Gating strategy is summarized in **Supplementary Figure S2**. Additionally, data sets from CD3^+^ cells of the negative control phenotypic characterization were downsampled to obtain 3000 events/fcs file. An unsupervised UMAP dimensional reduction was performed on FlowJo 10.5.2 using the default settings of a nearest neighbours of 15 and a minimum Euclidean distance of 0.5. The number of clusters was determined by X-Shift (Number nearest neighbors: 132, Euclidean distance metric) and FlowSOM and was applied to determine the level of expression of our proteins of interest in a total of 45 clusters in the CD3^+^ population. The abundance of each cluster was determined in HIV^+^ controllers and non-controllers and results were represented for each cluster as log_2_(%CD3^+^ cells in C/%CD3^+^ cells in NC). UMAP plot and clustering are summarized in **Figure S3**.

### IL-4, IL-21, and IFN-γ ELISpot

Mabtech ELISpot kits were used to count IFN-γ (capture clone 1-D1K, biotinylated clone 7-B6-1), IL-4 (capture clone IL-4I, biotinylated clone IL-4II) and IL-21 (capture clone MT216G, biotinylated clone MT21.3m) producing cells following manufacturer’s instructions. An additional assay, analogous to the *boosted Flow* technology was performed to enhance the sensitivity of detection by ELISpot. To that end, plates were coated with IL-4 and IL-21 capturing antibodies and IL-4 and IL-21 biotinylatated detection antibodies mixed, both at the concentrations recommended by the manufacturer’s, allowing us the identification of IL-4 and IL-21 producing cells. A total of 2x10^5^ PBMCs/well were stimulated for 48h at 37°C with peptide pools to which the individuals responded in the initial screening with CD8^+^ Tfc-like-reactive cells, using R10 as negative control in triplicates and PHA (25 µg/mL) as positive control. Spot forming cells (SFC) were counted using an automated Cellular Technology Limited (C.T.L.) ELISpot Reader Unit. The threshold for positivity was set as the highest of: a) 50 SFC/10^6^ PBMCs b) mean SFC in the negative controls plus 3 times standard deviations of the negative control wells or c) 3 times the mean of negative control well SFC/10^6^ PBMCs.

### Determination of Anti-Env IgM, IgA and IgG Humoral Response

HIV-1_NL4-3_ and HIV-1_BaL.01_ infected MOLT cells (50) were pre-incubated at room temperature for 30 minutes with plasma samples diluted 1/300 in PBS 1% BSA (Miltenyi Biotec). After three washes with PBS, the secondary antibodies: PE-F(ab)2 Goat anti-human IgG (Fc-specific); DyLight 649 Goat anti-human IgA and DyLight 488-F(ab)2 Goat anti-human IgM (Fcm5-specific) (Jackson Immunoresearch) were added separately and incubated for 15 minutes at room temperature. Stained cells were washed with PBS two times and fixed in 1% formaldehyde (Sigma-Aldrich). Samples were acquired in a BD Celesta cytometer (BD Biosciences) and the analysis was performed using FlowJo 10.5.2 software. HIV-1_NL4-3_ and HIV-1_BaL.01_ Envelope protein-specific IgM, IgA and IgG titers were correlated positively between them (**Figure S4**) suggesting that the difference in the signals is due to differences in surface stability of the strain-specific Env.

### IgG Avidity ELISA

To assess the avidity of gp120-specific IgG plasma antibodies of HIV controllers and non-controllers, we coated ELISA plates with HIV-1 (group M, subtype B, isolate BAL) gp120 Protein (SinoBiological) at 1 μg/ml in PBS overnight at 4°C. The following day, plates were blocked with PBS containing 1% BSA for two hours at room temperature. Plasma samples were diluted (range 1:1000 to 1:10000 depending on the IgG titer) and added to wells for two additional hours at room temperature. Each sample was added to four wells. Plates were then washed and incubated with either 2M guanidine HCl (two wells/sample) or PBS (two wells/sample) for 15 minutes at room temperature to allow disassociation. After washing, all wells were incubated for 30 minutes at room temperature with HRP-conjugated goat anti-human IgG (Jackson ImmunoResearch) at 1: 20,000. O-phenylenediamine dihydrochloride (OPD) (Sigma) was added to the plates and the enzymatic reaction was stopped with 2M of H_2_SO_4_. Signal was determined as the optical density at 492 nm, with noise correction at 620 nm. Avidity index of each sample was defined as the ratio between the mean signal obtained with and without guanidine treatment.

### Neutralization Assays

The neutralizing capacity of plasma was determined using a TZM-bl based pseudovirus neutralization assay as described elsewhere (51) using a panel of 5 pseudovirus, including HIV-1 strains classified as tier 1A (NL4-3), 1B (BaL.01), 2B (398F1) and 2A (TRO11 and AC10). Briefly, serial dilutions of heat inactivated (56°C 30 min) plasma samples were incubated with pseudoviruses in D10, DMEM supplemented with 100U/ml penicillin (Gibco), 100 µg/mL streptomycin (Gibco) and 10% heat-inactivated FBS (Invitrogen). Afterwards, they were added to TZM-bl cells treated with DEAE-Dextran (Sigma), plated at 10^4^ cells/well and cultured at 37°C for 48h. Then, 100µL of the supernatant was replaced with 100 µL of Britelite Luciferase Assay Substrate (Promega) and incubated for 1.5 minutes at room temperature. The cell-associated luciferase signal for each well was determined on an EnSight Multimode Plate Reader (Perkin Elmer). Neutralizing activity for each serum dilution was calculated as the percent of inhibition of viral entry compared to positive and negative controls, untreated infected cells and uninfected cells, respectively. Neutralization curves (percent viral entry inhibition versus plasma dilution) was generated using GraphPad Prism version 7 by fitting log_10_-transformed data with sigmoidal dose-response curve and IC_50_ was determined. Results were expressed as % of neutralizers in each group, neutralizing breadth (number of HIV-1 strains neutralized/number of HIV-1 strains tested * 100) and logIC50 (reciprocal plasma dilution) when data fitted a sigmoidal curve (HIV-1_NL4-3_ and HIV-1_BaL.01_).

### Antibody-Dependent Cellular Cytotoxicity (ADCC)

The ability of plasma to induce ADCC was determined by flow cytometry using a modified calcein-AM retention assay previously described (52). HIV_NL4-3_-infected target cells (CEM.NKR-CCR5 expressing the firefly luciferase downstream of the SIV long terminal repeat (LTR) promoter (53)) were washed and resuspended in 10 nM calcein-AM (BioLegend) diluted in R10 at a cell concentration of 10^6^ cells/mL for 30 minutes with intermittent shakes. Target cells were washed twice with R10 and 10^4^ target cells were cocultured in R10 supplemented with 10U/mL rh-IL-2 (Roche) in duplicates for 4 hours with the NK effector cell line [human CD16^+^ KHYG-1 (53)] at an E:T ratio of 5:1 in the presence or absence of serial dilutions (1:1000 to 1:256000) of HIV^+^ individuals’ plasma. After incubation, cells were washed with PBS plus 1% heat-inactivated FBS (Invitrogen) and 3x10^4^ cells were acquired on a LSRII BD instrument and analyzed on FlowJo 10.5.2 software. Results were normalized with a control condition in the absence of human plasma and lysis was calculated as the average of 100%-%calcein-AM^+^ cells in each duplicated condition.

### Statistical Analysis

GraphPad Prism version 7 for Mac OS X (La Jolla, CA) was used for statistical analyses. For comparison between group medians, non-parametric Mann-Whitney (MW) test was performed. Chi-square χ^2^ analysis was used to detect differences in the frequency of T cells with specific secreted cytokine patterns and number of samples with neutralizing activity. Correlation analysis was done applying the non-parametric Spearman r test. Statistical significance criteria were set at p<0.05.

## Results

### HIV-Specific T-Cell Responses With CD4^+^ Tfh-Like and CD8^+^ Tc2-Like and Tfc-Like Cytokine Polarization Profile Are Associated With Viral Control

**Figure 1:**
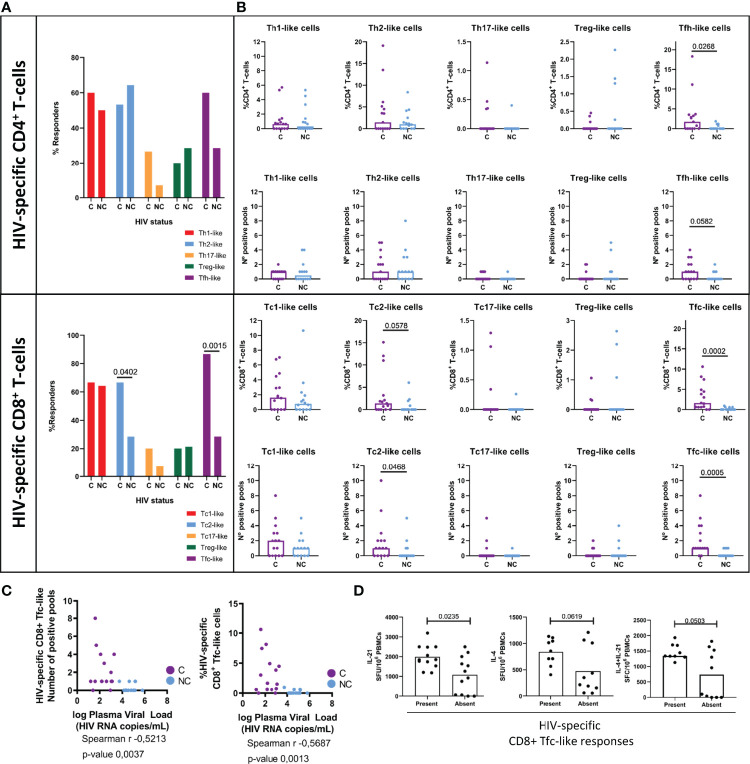
HIV-specific T-cell polarized responses. *Boosted Flow* screening of specific-T-cell responses against whole HIV proteome in a cohort of 15 HIV+ controllers (C, purple) and 14 HIV+ non-controllers {NC, light blue) revea led a highly-diverse set of polarized T-cell responses in terms of **(A)** number of responders **(B)** percentage of circulating HIV-specific T-cells (up) and number of reactive pools {down). **(C)** Negative correlation between plasma viral load and CDS+ Tfc-like responses to HIV measured in terms of breadth (l eft) and magnitude (right). **(D)** Inner capacity of total PBMCs to secrete T-follicular associated cytokines IL-21 (left scatter plot), IL-4 (middle scatter plot) and IL-21/IL-4 simultaneously (right scatter plot) upon non-specific PHA stimulation stratified by the presence (left bar) or the absence (right bar) of HIV-specific circulating Tfc-like responses. Statistical significance of differences in frequency of responders was evaluated by Chi-square test, between group medians by non-parametric Mann-Whitney test and correlation between Tfc-like responses and plasma viral load by Spearman correlation and its correspondent p-value. Statistical significance was set on p < 0.05.

To evaluate the presence of HIV-specific T-cells with alternative (non-IFN-γ dominated, i.e., Th1) cytokine polarization profiles, a comprehensive screening of HIV-specific cellular responses by *boosted flow* was performed. Total PBMCs from a cohort of 15 HIV^+^ controllers (C) and 14 HIV^+^ non-controllers (NC) were stimulated with a set of 17 peptide pools covering the entire HIV proteome. Observed signals in the flow cytometry data were expressed as the number of responders (% of individuals eliciting a specific polarized response), as well as breadth (number of reactive peptide pools) and magnitude (% of positive cells) of the response. While anti-HIV responses of type-1 CD4^+^ Th1-like cells and CD8^+^ Tc1-like cells were the most commonly detected responses, 86% (14 controllers and 11 non-controllers) of the tested 29 individuals also showed non-type-1-like responses (**Figure 1A**). Of these, five individuals (two controllers and three non-controllers) elicited exclusively responses with alternative polarization and no type-1 CD4^+^ or CD8^+^ T-cell responses. The dominant non-type-1 responses were type-2 in CD4^+^ (percentage of responders: CD4^+^ Th2-like cells 50% and CD8^+^ Tc2-like cells 47%) and follicular-like cells in the CD8 T cell compartment (percentage of responders: CD4^+^ Tfh-like cells 44%, CD8^+^ Tfc-like cells 53.5%). Type-3 polarized responses (percentage of responders: CD4^+^ Th17-like cells 16%, CD8^+^ Tc17-like cells 13%) and cells with regulatory functions (percentage of responders: CD4^+^ Treg-like 24%, CD8^+^ Treg-like 20%) were also observed in both groups (**Figure 1**).

An elevated magnitude (p= 0.0268) and breadth (p= 0.0582) of HIV-specific CD4^+^ Tfh-like cells (**Figure 1B**) was also observed in HIV^+^ controllers compared to non-controllers. CD8^+^ T-cell mediated responses with Tc2- and Tfc-like profiles were also more frequent (χ^2^ p=0.0402 and 0.0015, respectively) in HIV^+^ controllers (**Figure 1B**), who also showed significantly greater magnitude and breadth of CD8^+^ Tfc-like responses (magnitude, p=0.0005; breadth p=0.0002) (**Figure 1B**). The predominance of these CD8^+^ T-cell responses in HIV controllers was further evidenced by a negative correlation between the breadth and magnitude of HIV-specific CD8^+^ Tfc-like responses and the HIV plasma viral load (**Figure 1C**).

As the presence of CD8^+^ Tfc-like responses significantly differed between HIV controllers and non-controllers, we tested whether the acquisition of an anti-HIV CD8^+^ Tfc-like phenotype was related to a higher capacity of PBMCs to secrete T-cell follicular-related cytokines (IL-4, IL-21). Total PBMCs (8 HIV^+^ C and 14 HIV^+^ NC from the boosted flow study) were stimulated with the peptide pools that were found to trigger Tfc-like responses in the initial *boosted flow* screening. The screening was assessed individually or in combination (IL-4 and IL-21) by single-cytokine or combined ELISpot. In line with the boosted flow cytometry, 60% of stimulations induced IL-21 SFC and 30% induced IL-4 producing SFC. However, the combined detection of IL-4 and IL-21 increased the sensitivity of the ELISpot assay as responses against 80% of the pools were detected (**Figure S5**). Interestingly, individuals who showed the presence of HIV-specific CD8^+^ Tfc-like responses showed a significantly higher innate capability to secrete IL-21, but not IL-4 in response to an unspecific stimulus (PHA, **Figure 1D**).

**Figure 2:**
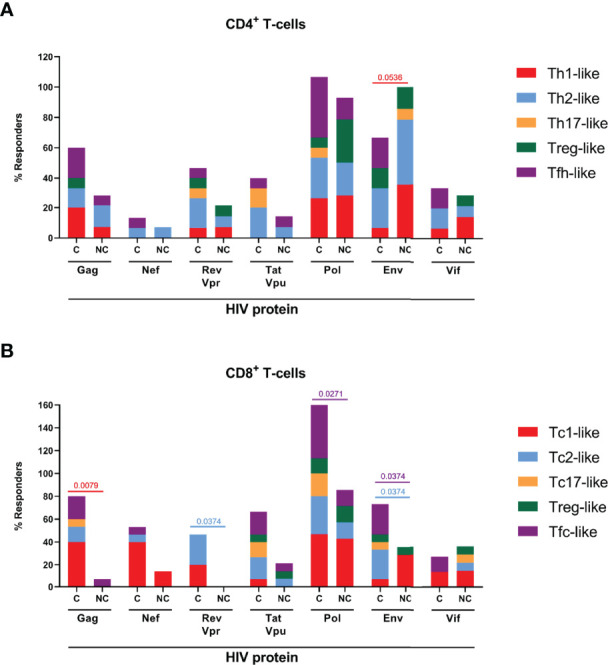
HIV protein-specific polarized T-cell responses. *Boosted Flow* screening of HIV protein-specific T-cell responses resulted into protein-dependent different polarized profiles in both CD4+ T-cells and CD8+ T-cells. The percentage of responders to each of the HIV-1 derived proteins is indicated in stacked bars for CD4+ **(A)** and CD8+ **(B)** T-cells. Statistically significant (p<O.O5) differences in protein-specific frequency of response between HIV+ controllers (C) and HIV+ non-controllers (NC) are indicated. Statistical significance was evaluated by Chi-square.

T-cell responses targeting different HIV proteins have been shown to exert different antiviral activity (54–57). This difference may be related to antigen presentation, viral sequence variability, but also to alterations in maturation and effector profile polarization in response to different viral proteins (58). Indeed, the analysis of T-cell responders stratified by HIV-derived protein revealed marked differences in the polarization profiles of responding T-cell populations (**Figure 2**) and these alterations were different in HIV controllers and non-controllers, linking viral protein specificity and effector function polarization to viral control. In particular and in line with previous studies, Th1-like CD4^+^ T-cell responses to Envelope (Env) were more frequent in HIV^+^ non-controllers compared to controllers (χ^2^ p=0.0536) (54, 55), while more CD4^+^ Tfh-like, Env-specific responders were preferentially, but not only, seen in HIV^+^ controllers (χ^2^ p=0.0772). HIV^+^ non-controllers also triggered more prominent Pol-specific Treg-like response (**Figure 2A**). In the case of CD8^+^ T-cells, statistically significant differences were observed for the frequency of type-1 responses to Gag-reactive responses in HIV controllers (χ^2^ p=0.0079), also in line with previous studies using single cytokine-based (IFN-γ) ELISpot analyses (46). Controllers also showed significantly higher number of Tc2-like responders upon Rev/Vpr or Env stimulation (χ^2^ p=0.0374) and Tfc-like upon Pol and Env stimulation (χ^2^ p=0.0271 and 0.0374, respectively, **Figure 2B**). When comparing the magnitude of responses, CD8^+^ Tc1-like Gag-specific (MW p=0.0169) and Tfc-like Pol-specific (MW p=0.0245) were stronger in controllers compared to non-controllers. Remarkably, accessory proteins (Rev, Vpr, Tat and Vpu) elicited more alternative effector functions than type-1 responses (**Figure S6**).

Polyfunctional analysis performed using the SPICE v6 software revealed T-cell responses with mostly single polarization profiles, indicating that responses might have matured into distinct polarization profiles, which are mutually exclusive, and supporting the specificity of the employed boosted flow analyses (**Figure S7**). In particular, Tfh and Tfc responses showed very little overlap with Th1 and Tc1 polarized responses, respectively, indicating that those subsets specifically produced IL-4/IL-21 but not Th1 cytokines such as IFN-γ/IL-2/TNF-α which have been observed in some earlier (murine) studies (43, 44). These results suggest that alternative HIV-specific T-cell profiling beyond conventional antiviral type-1 responses might be associated with viral control in a protein-dependent manner. The data also suggest that CD8+ Tfc-like cells might play a particularly important role in anti-HIV immune response.

### Circulating Type 2 and Follicular-Like T-Cells Show Different Memory Profiles in HIV^+^ Controllers and Non-Controllers

**Figure 3:**
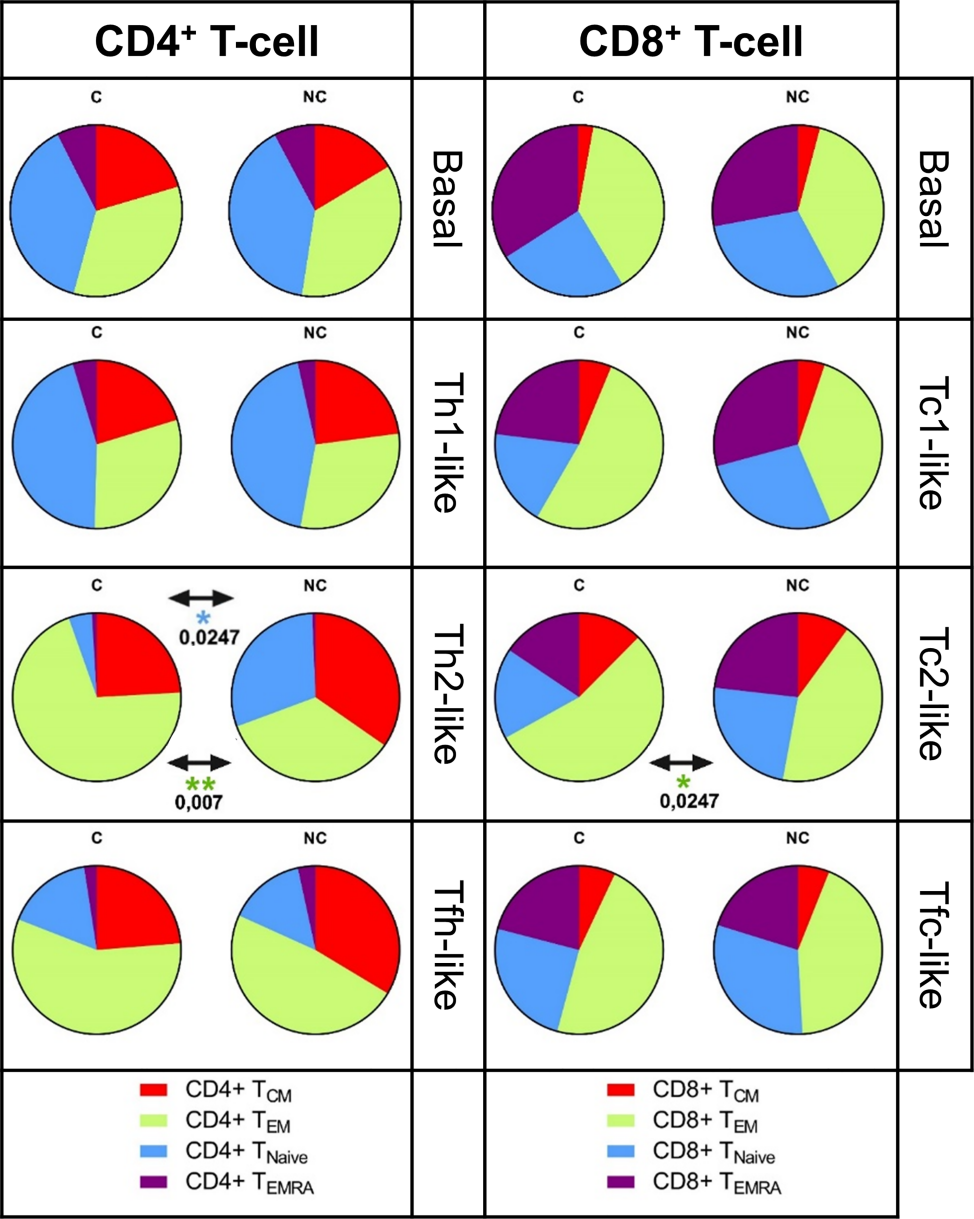
Memory phenotype of the HIV-specific polarized T-cell responses. The frequency of cells expressing memory markers CD45RA and CCR7 was evaluated in both HIV-specific polarized CD4+ and CD8+ T-ce lls. The percentage of basa l, Thl/Tcl, Th2/ Tc2 and Tfh/Tfc-like cells classified as TcM (CD45RA- CCR7+), TEM (CD45RA- CCR7-), TEMRA (CD45RA+ CCR7-) and TNaive (CD45RA+ CCR7+) are shown in pie charts. Statistical significance of the differences between HIV-specific memory phenotype in HIV+ controllers (C, left) and HIV+ non-controllers (NC, right) was evaluated by non-parametric Mann-Whitney test, statistical significance was set at p>O.OS, only significant results are shown.

To determine whether the polarization of the virus-specific effector T-cell responses and the apparent *in vivo* viral control were associated with the induction of specific T-cell differentiation and maturation, we determined CCR7 and CD45RA expression on HIV-specific CD4^+^ and CD8^+^ type-1, type-2 and follicular-like T-cells. No statistically significant differences were observed between unstimulated total CD4^+^ and CD8^+^ memory phenotypes in HIV^+^ controllers, compared to non-controllers (median percentage of CD4^+^ populations: T_CM_ 18%, T_EM_ 34.5%, T_EMRA_ 7.5% and T_Naive_ 40%; CD8^+^ T-cells: T_CM_ 3%, T_EM_ 37.8%, T_EMRA_ 29.9% and T_Naive_ 29.3%, **Figure 3**). In contrast, HIV-specific T-cells showed marked differences for T_Naive_ and T-memory subsets in the CD4^+^ Th2-like and Tfh-like, but not Th1 polarized cells. Of note, the levels of CD4^+^ T_EM_ contributing with Th2-like polarization were significantly higher (χ^2^ p=0.007) in HIV^+^ controllers, while T_Naive_ were more frequent in non-controllers (χ^2^ p= 0.0247). While differences in memory subset distribution were less evident for CD8^+^ T-cells, we observed a significant increase of T_EM_ Tc2 frequencies (χ^2 =^ 0.0247) and a reduction of T_Naive_ Tc2 populations, HIV-specific CD8^+^ cells in controllers compared to non-controllers. Altogether, these data revealed differences in the memory phenotype of HIV-specific type 2 T-cells that differ between HIV controllers and non-controllers.

### Expression of Cell Surface Specific Markers in Individuals Showing CD8^+^ Tc2- and Tfc-Like Responses

**Figure 4:**
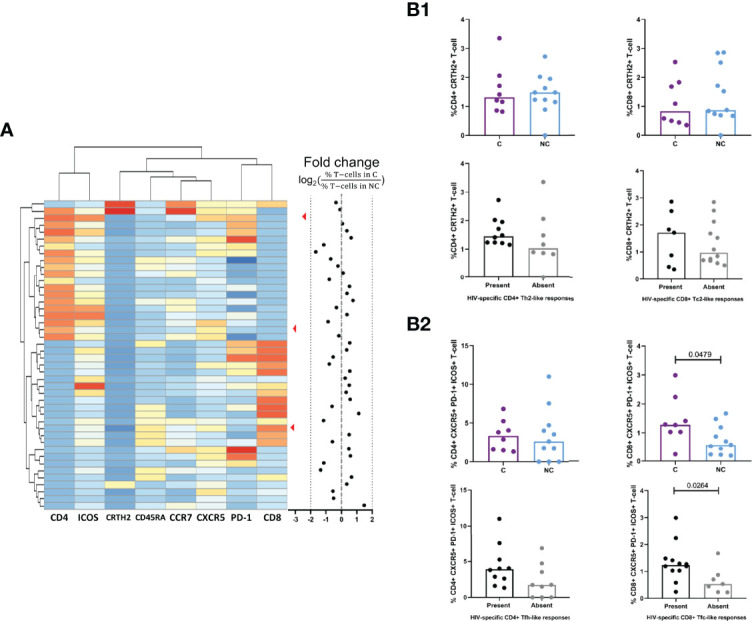
Comparison of CD4+ Th2 and Tfh and CDS+ Tc2 and Tfc subpopulations between controllers and non controllers. **(A)** Unsupervised clustering of CD4+ and CD8+ T-cell population based on the expression of Th2/Tc2 marker (CRTH2). Tfh/Tfc markers (CXCRS, PD-1, ICOS) and memory phenotype markers (CCR7, CD45RA) by FlowSOM. Results are expressed as a fold change between the presence of the clusters in HIV+ controllers (C, purple) compared to HIV+ non-controllers (NC, light blue). **(Bl)** Comparison of the percentage of CRTH2+ CD4+ Th2-cells (left) and CD8+ Tc2 cells (right) stratifying by HIV control status (above) or the presence or absence of HIV-specif ic Th2/Tc2 responses (below). **(B2)** Comparison of the percentage of CXCRS+PD-l+ICOS+ CD4+ Tfh-cells (left) and CD8+ Tfc cells (right) stratifying by HIV control status (above) and the presence or absence of HIV-specific Tfh/Tfc-like cytokine responses by boosted flow responses (below).

To further validate that the CD4^+^ and CD8^+^ type 2-like and follicular-like, HIV-specific T-cells identified by our boosted flow cytometry approach expressed traditional markers of Th2/Tc2 or Tfh/Tfc, we determined expression of CRTH2 (59, 60) for Th2/Tc2 or CXCR5, PD-1 and ICOS for Tfh/Tfc (**Figure S4**) by flow cytometry. An unsupervised analysis by X-Shift yielded a total of 45 clusters based on the expression of CD4, CD8, CCR7, CD45RA, CRTH2, CXCR5, PD-1 and ICOS. A heatmap was generated based on the level of expression (MFI) of each marker. The contribution of each cluster inside the CD3^+^ population was used to compare controller and non-controller status as a fold change. Clusters contributing with a 2-fold change to the CD3^+^ population (%) were included in the analysis (**Figure 4A**). Three different clusters, one corresponding to CD8^+^ T-cells and two belonging to CD4^+^ were overrepresented in HIV^+^ non-controllers. Of those, one cluster of CD4^+^ Tfh cells expressing ICOS, CXCR5 and PD-1. The other two clusters (one CD4^+^ and one CD8^+^) expressed high levels of CXCR5 but lacked the expression of PD-1 and ICOS. We then explored by manual supervised gating the potential relationship between the presence of circulating CRTH2+ Th2/Tc2, circulating CXCR5^+^ Tfh/Tfc populations and HIV control (**Figure 4B**). These analyses did not reveal any differences in the levels of CD4^+^ Th2 cells expressing CRTH2, or CD4^+^ Tfh cells expressing CXCR5, PD-1 and ICOS. Similarly, no difference in CRTH2 expressing Th2 CD8^+^ T-cells was observed. However, CD8^+^ CXCR5^+^PD-1^+^ICOS^+^ T-cells were significantly elevated in HIV^+^ controllers (MW, p-value=0.0479) and also in those individuals capable to elicit HIV-specific CD8^+^ Tfc-like cytokine responses, as seen within the *boosted flow.* These results provided evidence of deficient expression of follicular markers in peripheral Tfc-like cells among HIV non-controllers that could impact their function regarding B-cell interaction and antibody maturation.

### Higher Levels of CD8^+^ Tfc-Like Cells in HIV^+^ Controllers Are Associated With Maturation of the Humoral Immune Response and Antibody Effector Functions

**Figure 5:**
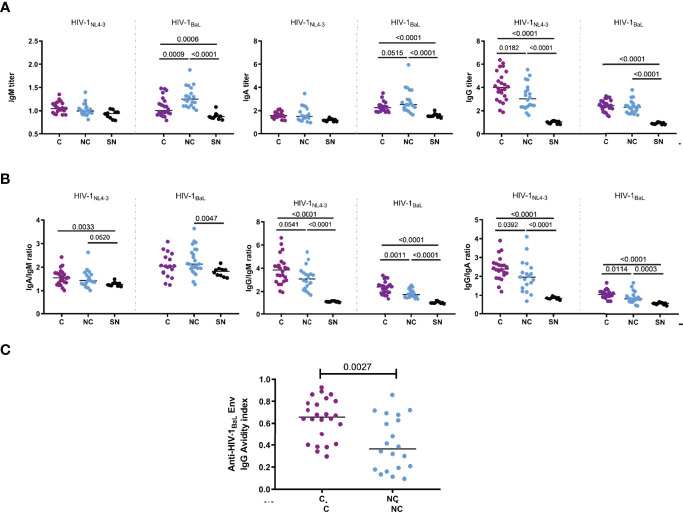
Titers of anti-HIV Env lgA, lgG and lgM. Titers and affinity were compared between controllers (N=24, C, purple dots) and noncontrollers (N=20, NC, light blue dots) and HIV seronegatives (N= 8, SN, black dots). **(A)** Plasma dilution titers of anti-HIV Env lgA, lgG and lgM were determined with MOLT-4 cell lines expressing HIV-1NL4_3 and HIV-18AL Env in the surface. **(B)** lsotype class switching extend was estimated by the ratio of anti-HIV Env lgA/IgM, lgG/IgM and lgG/IgA in each individual. **(C)** Avidity index of HIV-lsaL Env-specific lgG. Statistical significance (p< 0.05) was evaluated by applying non-parametric Mann-Whitney test.

CD4^+^ Tfh-like cells have been shown to be required for the induction of effective humoral immunity. Thus, we assessed whether HIV-specific CD4^+^ and especially also CD8^+^ Tfc-like cells, were related with antibody isotype switching, neutralization capacity and ADCC effector function. To this end, we first determined plasma titers of HIV-1_BaL.01_ and HIV-1_NL4-3_ Env-specific IgA, IgM and IgG in 24 HIV^+^C, 20 HIV^+^ NC and 8 HIV^-^. Uncontrolled HIV infection was associated with increased median IgA (p=0.0515) and IgM titers (p=0.0009), while HIV^+^ controllers’ plasma contained higher titers of IgG (p=0.0182, **Figure 5A**). IgG/IgM and IgG/IgA ratios, reflecting isotype class switching towards IgG, were elevated in HIV controllers (**Figure 5B**). Furthermore, the avidity of the anti-HIV-1_BaL.01_ gp120 specific IgG response was significantly higher in plasma from HIV^+^ controllers (MW, p=0.0027) (**Figure 5C**).

**Figure 6:**
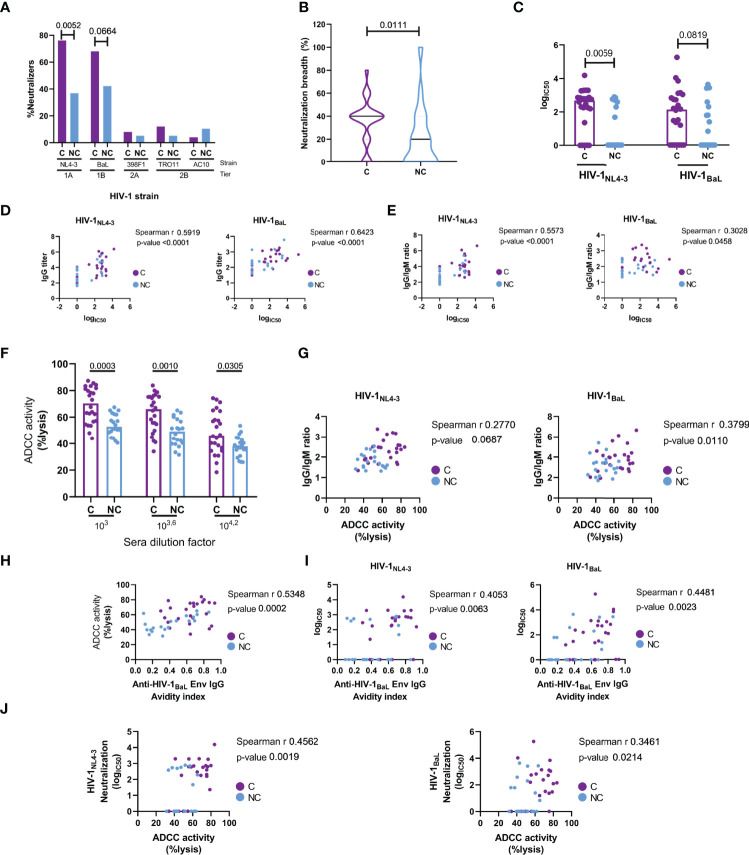
Antibody mediated anti-HIV functional responses. Responses are compared between controllers (**(C)**, purple) and non-controllers (NC, light blue). **(A)** Percentage of individuals capable to neutralize a panel of 5 HIV-1 pseudoviruses, including 5 strains from tier 1A (NL4-3), 1B (BaL), 2A (398F1) and 2B (TROll and AClO). **(B)** Neutralizing breadth (left) in HIV+ controllers **(C)** and non-controllers (NC) calculated as: number of pseudovirus neutralized/number of pseudovirus evaluated (5) *100. **(C)** IC50 neutralizing capacity of HIV+ controllers **(C)** and noncont rollers (NC) against HIV-1NL4_3 (left) and HIV-18aL (right). **(D)** Spearman correlation of logiC50 neutralizing capacity with lgG titers. **(E)** Correlation of logiC50 neut ralizing capacity with lgG/IgM ratio. **(F)** Ant ibody-dependent ce llular cytotoxicity (ADCC) induced by t he presence of serial sera dilutions from cont rollers **(C)** and non-cont rollers (NC). **(G)** Correlation between ADCC at 1:104·2 serum dilution with lgG/IgM ratio. **(H)** Correlation between ADCC at 1:10^4.2^ serum dilution with avidity index. **(I)** Correlations between IC50 neutralizing capacity against HIV-1NL4_3 (left) and HIV-18aL (right) with avidity. **(J)** Correlation between ADCC at 1:10^4.2^ serum dilution with neutralization log(IC50). Statistical significance of the presence of neutralizing activity **(A)** in both groups was evaluated by Chi-square, while comparison of medians (B, C and F) was det ermined by non-parametric Mann-Whitney test . Correlations were performed by non-parametric Spearman r test, rho and p-values are displayed in the correspondent plots.

Neutralization capacity and ADCC activity were determined as measures of antibody-mediated effector function. As shown in **Figure 6A**, neutralization capacity was assessed against a panel of five HIV-1 pseudovirus, including strains from tiers 1A (NL4-3), 1B (BaL.01) and 2 (398F1, TRO11 and AC10). Among HIV controllers, a significantly higher proportion of individuals (76%) were able to neutralize HIV-1_NL4-3_ compared to NC (37%, χ^2^ p=0.0052). A similar trend was observed for neutralization of the HIV-1_BaL.01_ isolate (68% C vs 42% NC, χ^2^ p=0.0664). Neutralization breadth was also significantly increased in controllers compared to non-controllers (median: 40% and 20% respectively; χ^2^, p=0.0111) (**Figure 6B**). The neutralization capacity against HIV-1_NL4-3_ strain was also significantly elevated in controllers (MW, p=0.0059) and a trend (MW, p=0.0819) for neutralization of HIV-1_BaL.01_ was observed as well (**Figure 6C**).

**Figure 7:**
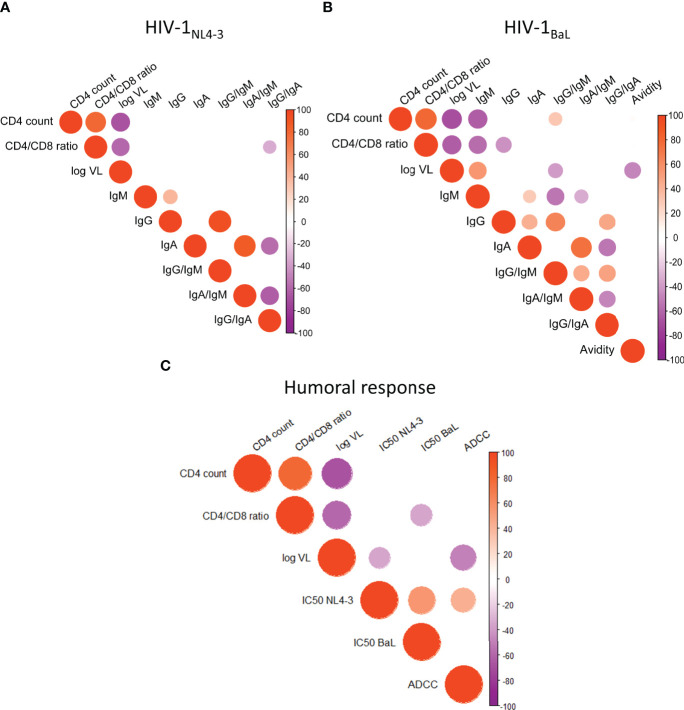
Humoral anti-HIV functional responses link with clinical parameter's. **(A)** Correlation between anti-HIV-1NL4_3 antibody titers and CD4 count, CD4/CD8 ratio and plasma viral load. **(B)** Correlation between anti-HIV-lBaL antibody titers and CD4 count, CD4/CD8 ratio and plasma viral load. **(C)** Correlation between humoral functional response (HIV-1NL4_3 and HIV-lBad neutralization activity, ADCC and CD4 count, with CD4/CD8 ratio and plasma viral load. Correlations were performed by non-parametric Spearman r test. Positive correlations are indicated in red while negative correlations in purple, color intensity in proportional to Spearman's rho and circle size is related to the p-value when significant.

Along with neutralization, more potent ADCC activity was observed in controllers’ plasma compared to non-controllers. We also observed significant positive correlations between ADCC capacity (1:10^3.6^ dilution) and neutralization IC50 for HIV-1_NL4-3_ (rho= 0.4562, p=0.0019) and HIV-1_BaL.01_ (rho= 0.3461, p=0.0214, **Figure 6J**). These associations were driven by the levels of IgG and IgG avidity in the plasma samples, as we found Env-specific IgG (but not other isotypes) titers to be positively correlated with logIC50 for HIV-1_NL4-3_ (rho= 0.5919, p<0.0001) and HIV-1_BaL.01_ (rho= 0.6423, p<0.0001) (**Figures 6D–F**) and avidity to be positively correlated with ADCC capacity (rho=0.5348, p=0.0002) (**Figure 6H**) and neutralization against HIV-1_NL4-3_ (rho= 0.4053, p=0.0063) and HIV-1_BaL.01_ (rho= 0.4461, p=0.0023) (**Figure 6I**). IgG/IgM ratios also correlated with neutralization of HIV-1_NL4-3_ (rho=0.5573, p<0.0001) and HIV-1_BaL.01_ (rho= 0.3028, p=0.0458). A similar relation of the IgG/IgM ratio was seen with the ADCC capacity not only with the IgG/IgM ratio for the HIV-1_NL4-3_ Env (rho= 0.2770, p =0.0687), which is the same HIV strain expressed by the CEM.NKR-CCR5 ADCC target cells, but also with the IgG/IgM ratio for the HIV-1_BaL.01_ Env (rho= 0.3799, p =0.0110 - **Figure 6G**). To further explore a possible relationship between the humoral immune response with HIV control, antibody titers and functional responses were correlated with three clinical parameters (CD4 count, CD4/CD8 ratio and plasma viral load). Anti-HIV-1_BaL.01_ IgM titers showed a negative correlation with CD4 count (rho= -0.6352, p<0.0001), CD4/CD8 ratio (Spearman r rho=-0.5628, p<0.0001) and a positive correlation with plasma viral load (Spearman r rho=0.4177, p= 0.0048) (**Figure 7**), in line with the elevated IgM levels seen in HIV non-controllers. Furthermore, plasma viral load was also negatively correlated with IgG/IgM ratio (rho= -0.3094, p=0.0410), plasma avidity (rho=-0.4716, p=0.0012), neutralizing capacity against HIV-1_NL4-3_ (rho=-0.3989, p=0.0073) and ADCC activity (rho= -0.4863, p=0.0008; **Figure 7**). Our results demonstrate a higher extent of anti-Env IgG with increased avidity and a decrease of IgM antibodies in individuals with natural control that was related to a higher neutralizing capacity and elevated ADCC activity.

### HIV-Specific CD8^+^ Tfc-Like Responses Are Linked to Antibody Isotype Class Switching and Humoral Function

**Figure 8:**
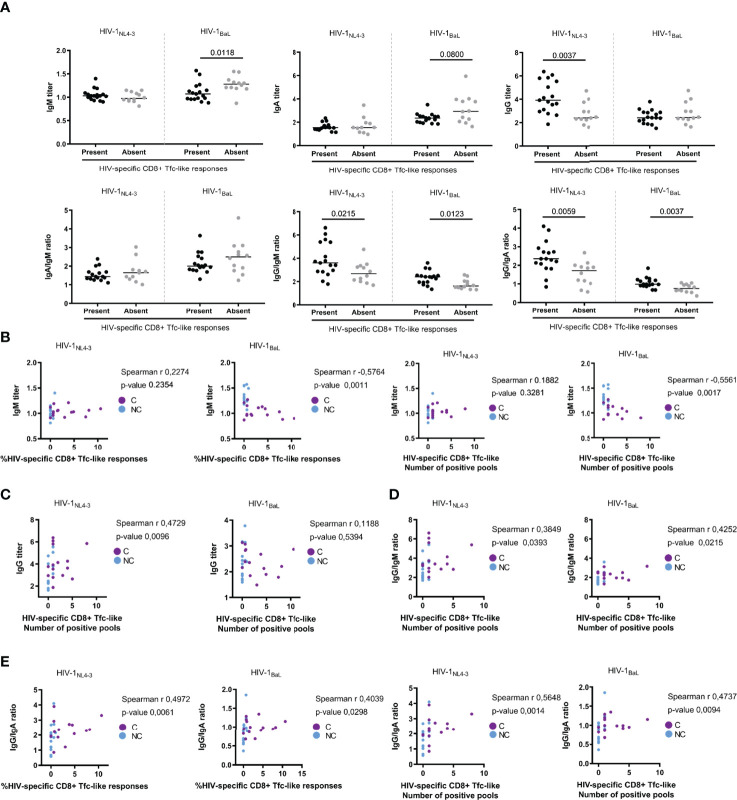
Link between HIV-specific CDS+ Tfc-like responses and humoral responses. **(A)** Samples were stratified by the presence (n=17) or the absence (n=13) of HIV-specific CD8+ Tfc-like responses and titers of lgA, lgG and lgM were compared, as well as the lgA/IgM, lgG/IgM and lgG/IgA ratios. **(B)** Correlation between anti-HIV-1NL4_3 (left) and HIV-lsaL (right) lgM titers with CD8+ Tfc-like magnitude (left) and breadth (right). **(C)** Correlation between anti-HIV-1NL4_3 (left) and HIV-lsaL (right) lgG titers with CD8+ Tfc-like breadth. **(D)** Correlation between anti-HIV-1NL4.3 (left) and HIV-lsaL (right) lgG/IgM ratio with CD8+ Tfc-like breadth. **(E)** Correlation between anti-HIV-1NL4_3 (left) and HIV·lsaL (right) lgG/IgA ratios with CD8+ Tfc-like magnitude (left) and breadth (right). Statistical significance of the comparison were performed by non-parametrical Mann-Whitney test while non-parametric Spearman r correlations were applied.

Our data show HIV-specific CD8^+^ Tfc-like responses to be elevated in HIV controllers, as well as to be linked to isotype ratios and IgG titers. Thus, we tested directly how the presence of CD8^+^ Tfc-like cells was related to these beneficial markers of the humoral immune response. Plasma from individuals with detectable CD8^+^ Tfc-like cells demonstrated increased titers of IgG to HIV_NL4-3_ Env (p=0.0037) and decreased titers of IgM HIV-1_BaL.01_ Env (p=0.0118). This finding resulted in a significantly higher IgG/IgM ratio for both viral isolates (HIV-1_NL4-3_ MW p=0.0215, HIV-1_BaL.01_ MW p=0.0123) and reflected increased antibody isotype class switching when compared to individuals with no detectable CD8^+^ Tfc-like responses. When measuring the balance of IgG with IgA levels, the results showed a clear dominance of IgG mediated antibody activity when CD8^+^ Tfc-like responses were present (HIV-1_NL4-3_ MW p=0.0059, HIV-1_BaL.01_ MW p=0.0037) (**Figure 8A**). In addition, we observed that the magnitude and breadth of the CD8^+^ Tfc-like response correlated negatively with anti-HIV-1_BaL.01_ IgM (Magnitude: r = -0.5764, p= 0.0011; Breadth: r = -0.5561, p= -0.0017 - **Figure 8B**) and positively with anti-HIV-1_NL4-3_ IgG (Breadth: rho=0.4729, p = 0.0096 - **Figure 8C**) and IgG/IgM ratio for both strains (HIV-1_NL4-3_: rho= 0.3849, p = 0.0393; HIV-1_BaL.01_: r = 0.4252, p = 0.0215 – **Figure 8D**). Interestingly, IgG/IgA ratio also positively correlated with Tfc-like magnitude and breadth, showing a preferential isotype switching towards IgG over IgA in those individuals who showed an expansion of HIV-specific CD8^+^ Tfc-like cells (**Figure 8E**).

## Discussion

Polarization of HIV-specific T-cell immunity has been suggested to play a role in virus control. Besides the original descriptions of Th1 and Th2 profiles in HIV infection (27, 28), some previous studies found the Th17/Treg balance to influence HIV control as well (31, 32). However, little new insights have been gained on alternative polarization profiles and HIV control and disease course (61). Using a novel, *boosted* flow cytometry approach, we here analyzed alternative polarization profiles of virus-specific CD4^+^ and CD8^+^ T-cell subsets and determined their role in viral control and markers of humoral immunity. Our results identify a HIV-specific CD8^+^ Tfc-like cell subset in individuals with a natural control of infection and links the strength of these responses to the antibody isotype switching to IgG, higher anti-Env IgG titers and superior neutralization capacity and ADCC activity. These results point towards a potential link between a virus-specific CD8^+^ T follicular cell population and the humoral immunity to HIV, that may provide guidance for future vaccine development.

The contribution of type 1 CD4^+^ and CD8^+^ T-cell responses to HIV control has been very well studied, especially those mediated by cells producing IFN-γ. However, IFN-γ production is only a part of the T-cell effector function profile and different cytokines are orchestrating various arms of the host immunity. Yet, only few studies have analyzed CD4^+^ and CD8^+^ T-cell subsets beyond type 1 responses in relation to viral control (25). To some degree, this may be due to the fact that these alternative subsets may be of low frequency among the virus-specific T-cell in peripheral blood. In addition, these cells may not produce large amounts of individual, profile-defining cytokines, thus escaping detection by current standard intracellular staining flow cytometry-based assays. *Boosted* flow cytometry assay fills this critical gap by pooling multiple cytokine signals into the same detection channel, thus adding up individuals signals and pushing the staining intensity above the limits of detection. The first version of the *boosted* flow allowed for the detection of alternative effector functions in virus-specific T cells in HIV controllers and in highly-exposed individuals that remained HIV negative (47, 62). Here, applying a boosted flow cytometry panel with improved coverage, allowed us to identify HIV-specific T-cell responses beyond type 1 in 86% of the individuals that would have been missed by single cytokine detection. Among them, CD4^+^ Tfh, CD8^+^ Tc2 and especially Tfc-like responses were more frequent and showed greater magnitude and breadth in individuals with natural control of HIV infection. Of these subsets, CD8^+^ Tfc-like responses showed particularly strong associations with parameters of virus control and correlated directly with markers of humoral immune responses to HIV. These responses were detected by boosted flow cytometry but not when detecting IL-4 and IL-21 in separate channels by conventional ICS (data not shown). Similarly, few IL-4 or IL-21 secreting T-cells were identified by single cytokine ELISpot, unless the detection of both cytokines was combined in the assay. Of note, polyfunctional analysis showed that most of the pool-specific responses showed a single polarization profile, indicating that mixed T-cell polarization profiles may be infrequent (14). These CD8^+^ Tfc have just recently been identified and share characteristics with both CD4^+^ Tfc and CD8^+^ Tc1 cells. As CD4^+^ Tfh T-cells, they maintain a self-renewal phenotype and the ability to promote B-cell maturation and isotype class switching. In addition to the increased cytotoxic activity and ability to migrate to the follicles, CD8^+^ Tfc support their potential role in controlling HIV replication and in reducing viral reservoir (22), which may be of significant interest for HIV immunotherapeutic strategies.

The present data indicate that viral proteins trigger different polarization profiles and that these protein-specific T-cell subsets can be related to HIV control, as it has been described for Tc1 and Th1 responses (54–56). In line with these results, we observed an increase in CD4^+^ Th1-like responses to Env in HIV^+^ non-controllers, while controllers had stronger CD8^+^ Tc1-like responses to Gag. Additionally, CD8^+^ Tfc responses of higher magnitude to HIV Pol and Env were observed in controllers. A similar observation had already been reported for CD4^+^ Tfh responses, which showed a different protein specificity compared to the classical CD4^+^ Th1-like with a lower trend to respond to Gag stimulation over Env (38). Similarly, accessory/regulatory HIV-proteins have been reported to be targeted by Tc1, but not by Th1. Here, we also show that accessory proteins were mainly targeted by Th2 polarized CD8^+^ T-cell subsets, which usually secrete lower cytokine levels and could be largely missed when using standard intracellular cytokine staining. Peptide HLA binding affinity, protein expression kinetics, protein levels and antigen processing preferences have been proposed as potential mechanisms for these different polarization profiles triggered by different HIV proteins (58).

Analysis of the surface expression of CCR7 and CD45RA memory markers revealed differences among Th1, Th2 and Tfh polarized T cells. In particular, Th2 and Tfh showed increased T_EM_ (CD45RA^-^ CCR7^-^) subpopulations and decreased T_Naive_ (CD45RA^+^ CCR7^+^) compared to Th1 polarized CD4^+^ T cells. Also, an increased percentage of CD4^+^ T_Naive_ cells with a Th2 profile in HIV^+^ non-controllers, along with a significant reduction of T_EM_ in CD4^+^ Th2 and CD8^+^ Tc2, respectively was observed, indicating a higher capacity to express type 2 cytokines in HIV controllers. These results suggest that although T_EM_ Th2 and Tc2 populations persist long term *in vivo* and have been associated with severe asthma (63, 64), their role in chronic HIV infection might be beneficial as their presence is associated with viral control.

CXCR5^+^ CD8^+^ T-cells have been described as a major source of IL-21 and have been shown to be related to lower HIV viral loads (65). Here, we observed higher numbers of HIV-specific CD8^+^ Tfc-like responses in controllers and an increased innate IL-21 and IL-4 secretion capacity in total PBMCs together with a higher proportion of circulating CD8^+^ Tfc (CXCR5^+^PD-1^+^ICOS^+^) in individuals showing Tfc-like responses. Our unsupervised analysis of circulating Th2/Tc2 and Tfh/Tfc populations also revealed a higher proportion of peripheral CD4^+^ CXCR5^+^ PD-1^+^ ICOS^+^ cells in non-controllers, as suggested in previous studies (41). We also observed more prominent CXCR5^+^ CD4^+^ and CD8^+^ T-cell populations in non-controllers that lacked the expression of ICOS or/and PD-1. Since these surface proteins are important for the interaction with B-cells, the reduced expression may explain the smaller extent of antibody isotype class switching and maturation in these individuals (20). This fact might represent an innate predisposition to generate CD8^+^ Tfc responses upon HIV infection in some individuals, with potential consequences on antibody isotype switching and viral control. Indeed, HIV^+^ non-controllers showed differences in the anti-Env antibody response characterized by a reduction in IgG and an increase in IgM isotype use. This fact is in line with reduced antibody switching to IgG and diminished avidity in HIV^+^ non-controllers compared to controllers, estimated as the IgG/IgM and IgG/IgA ratios and avidity index, respectively. Chronic HIV infection has been associated with the dysregulation of the germinal center reaction, impairing the generation of memory B-cells (66). Thus, the presence of higher levels of reactive Tfc-like cells could reflect a better-preserved germinal center interaction in individuals with natural control of HIV infection (67, 68). However, peripheral blood Tfc-like responses might not properly reflect germinal center interaction and partially explain the lack of correlation between avidity and Tfc or Tfh-like responses against HIV (69).

Interestingly, the correlation between HIV-specific Tfc responses and increased IgG/IgM ratio suggest a role of CD8^+^ Tfc-like cells in the maturation of the antibody response in HIV infection. Furthermore, humoral effector functions, determined as the neutralization capacity and ADCC activity, were more robust among HIV controllers and correlated with isotype class switching related to Tfc responses and avidity. Neutralization and ADCC capacity correlated with each other and with the IgG/IgM ratios and IgG avidity, in line with previous studies (70, 71). Interestingly, the neutralization capacity was also related to IgA/IgM titers. Mucosal IgA has been reported to neutralize HIV (72), but the role of this isotype in plasma has been less explored, although some studies reported a correlation between serum IgA titers and neutralization capacity (73).

Our study has a number of limitations regarding the evaluation of humoral effector functions and isotype switching. BCR sequencing would be required to confirm that the higher IgG/IgM ratio is indeed due to a higher number of specific B-cell clones with isotype class switching. We have not measured specific IgG subtypes with different functional effects which generation may be impacted by T helper polarization. Additional studies, such as CD40L expression on Tfc cells and the distribution of B cell subsets will possibly proof insightful to further understand the Tfc/B cell crosstalk in the context of chronic HIV-1 infection. Notwithstanding these limitations, we founded that the strength and effector functions of the humoral response was related to CD8^+^ Tfc responses, but not Tfh or other T helper functions, pointing towards a central role of this subset in orchestrating the humoral immunity to HIV.

Thanks to the extensive coverage of different effector functions, their detection by accumulating signals using the boosted flow cytometry, their integration with antibody isotype class switching to IgG and the associated humoral responses analysis, we identified Tfc responses that were associated with the clinical course of HIV infection. The presence of these cells was associated with lower viral load and preferential antibody isotype class switching to IgG which are associated with increased neutralizing and ADCC activity. IgM titers were observed to be detrimental and are related not only to higher plasma viremia, but also with the loss of CD4^+^ T-cells. The identification of virus-specific T-cell responses that can orchestrate the humoral responses and can infiltrate tissues of viral reservoir is of potential interest for vaccine development and, leveraging the potential plasticity of polarized T-cell populations, may open the door for future therapeutic interventions as has been postulated, for instance, to improve anti-cancer immunity (74).

## Data Availability Statement

The original contributions presented in the study are included in the article/supplementary material. Further inquiries can be directed to the corresponding author.

## Ethics Statement

The studies involving human participants were reviewed and approved by the Ethics Committee of the Hospital Universitari Germans Trias i Pujol in Badalona, Spain (PI-18-011). All subjects gave written informed consent in accordance with the Declaration of Helsinki. The patients/participants provided their written informed consent to participate in this study.

## Author Contributions

Conceptualization: LR-M, MR-R, CB, and AO. Methodology: LR-M, FT-F, MR, FC, JC, MR-R, and AO. Investigation: LR-M, FT-F, and MR. Visualization: LR-M, CB, and AO. Funding acquisition: CB, AO, and DO’C. Project administration: CB, BM, and AO. Supervision: JB, MR-R, CB, and AO. Writing – original draft: LR-M and AO. Writing – review & editing: All authors contributed to the article and approved the submitted versions.

## Funding

The present study was supported by grant PI17/01465 (AO) from the Instituto de Salud Carlos III, co-financed by the Fondo Europeo de Desarrollo Regional (FEDER) “Una manera de hacer Europa” and funding from the European Union’s Horizon 2020 research and innovation program under grant European AIDS Vaccine Initiative 2020 (EAVI2020) #GA681137 (CB), “La Caixa” Foundation under the project code HR17-00199 and by a grant from the National Institute of Allergy and Infectious Diseases under award number PO1-AI131568. CB is a Senior ICREA research professor.

## Conflict of Interest

Author CB was employed by company AELIX Therapeutics. MR-R and CB are co-inventors of the “Boosted flow” technology, which is protected under patent application US9709577B2.

The remaining authors declare that the research was conducted in the absence of any commercial or financial relationships that could be construed as a potential conflict of interest.

## Publisher’s Note

All claims expressed in this article are solely those of the authors and do not necessarily represent those of their affiliated organizations, or those of the publisher, the editors and the reviewers. Any product that may be evaluated in this article, or claim that may be made by its manufacturer, is not guaranteed or endorsed by the publisher.
