# Supplementary material for: T-Follicular-Like CD8+ T Cell Responses in Chronic HIV Infection Are Associated With Virus Control and Antibody Isotype Switching to IgG
Source: Front Immunol. 2022 Jun 15;13:928039. doi: 10.3389/fimmu.2022.928039 (PMC9241491; doi:10.3389/fimmu.2022.928039)
Supplement: Supplementary file 1 [file Table_1.docx]

**Supplementary Table 1. Clinical characteristics of the participants in the study**
