# Supplementary figures and images for: T-Follicular-Like CD8+ T Cell Responses in Chronic HIV Infection Are Associated With Virus Control and Antibody Isotype Switching to IgG
Source: Front Immunol. 2022 Jun 15;13:928039. doi: 10.3389/fimmu.2022.928039 (PMC9241491; doi:10.3389/fimmu.2022.928039)

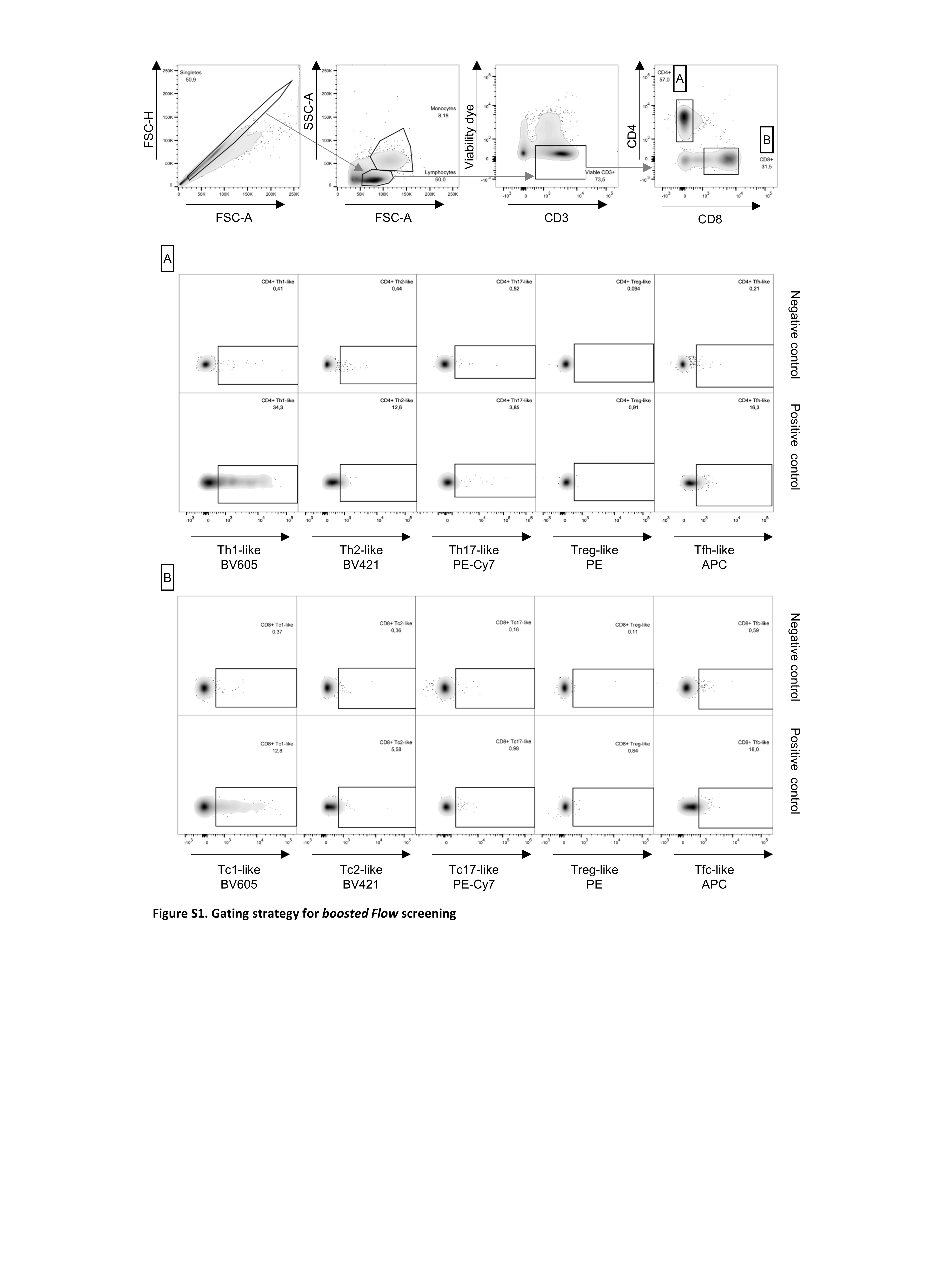

Supplement: Supplementary file 2 [file Image_1.tif]

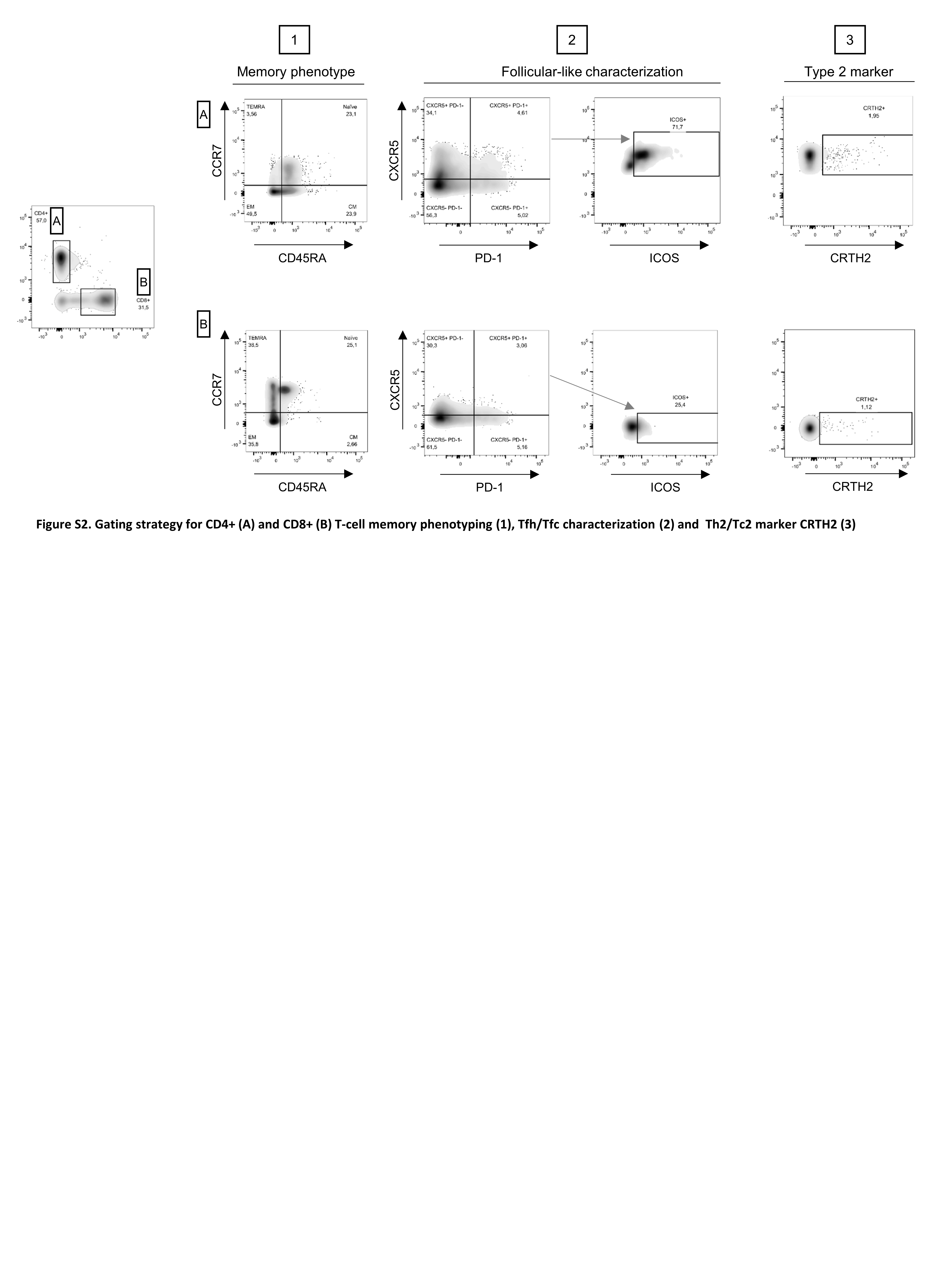

Supplement: Supplementary file 3 [file Image_2.tif]

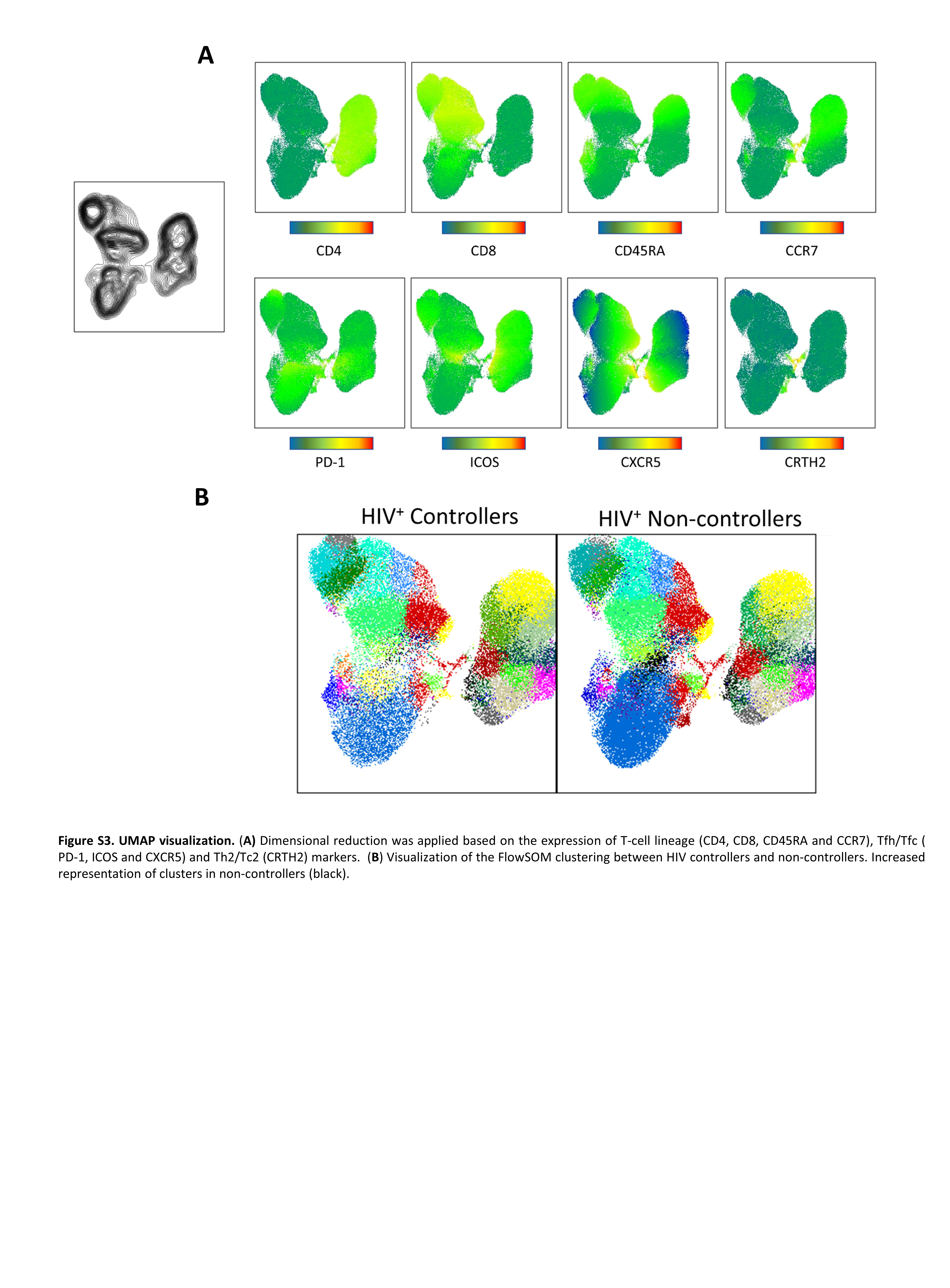

Supplement: Supplementary file 4 [file Image_3.tif]

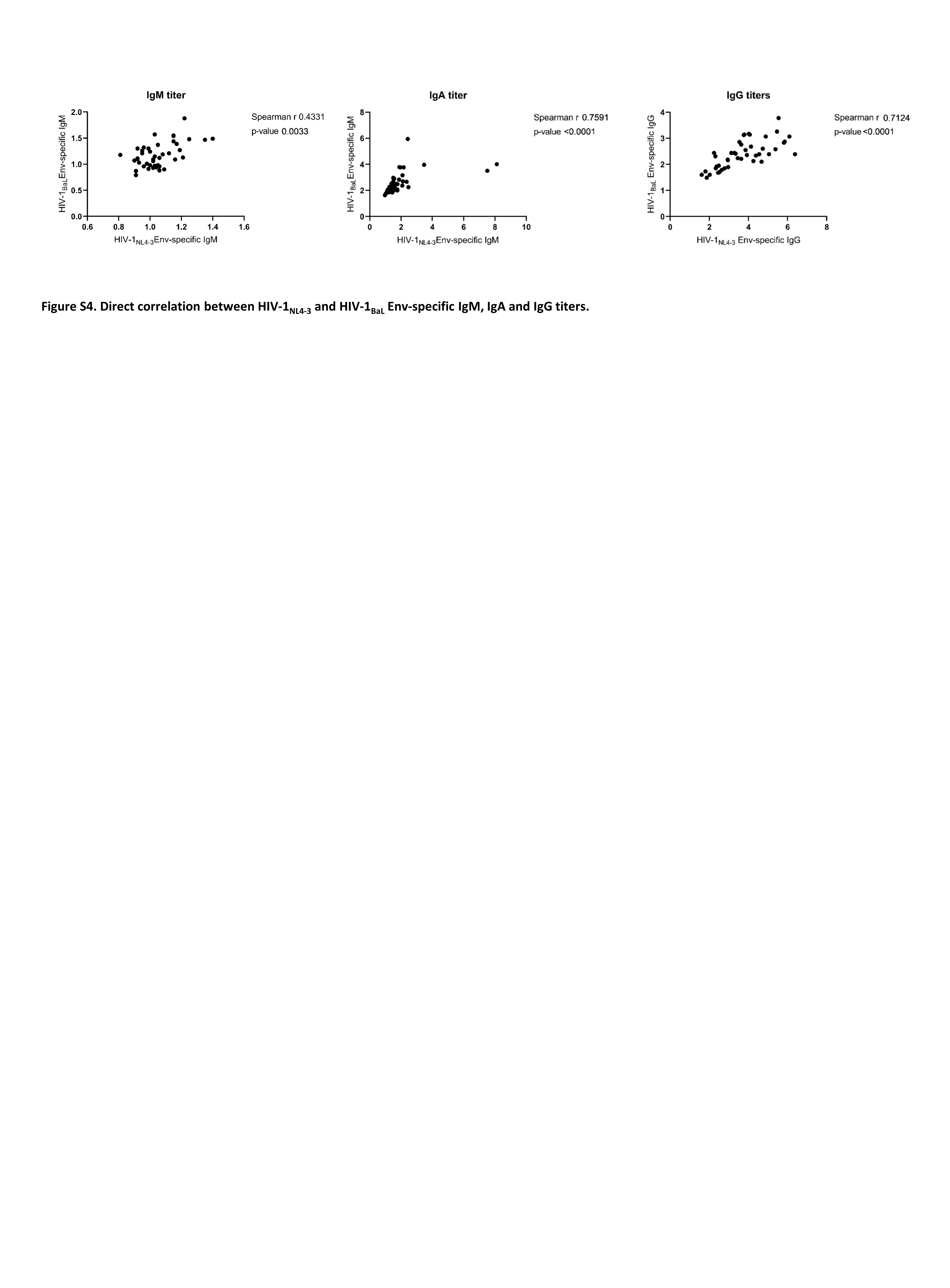

Supplement: Supplementary file 5 [file Image_4.tif]

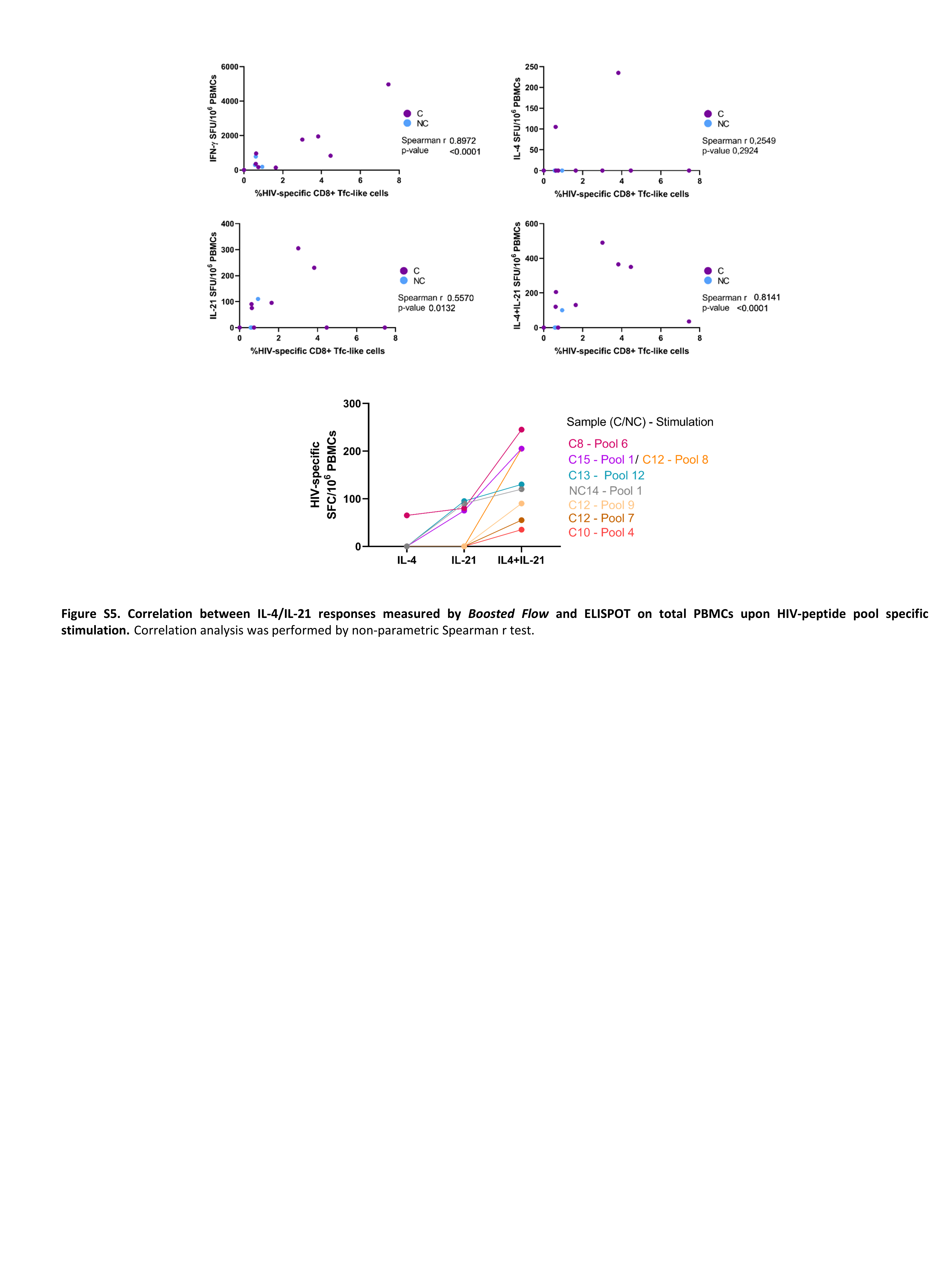

Supplement: Supplementary file 6 [file Image_5.tif]

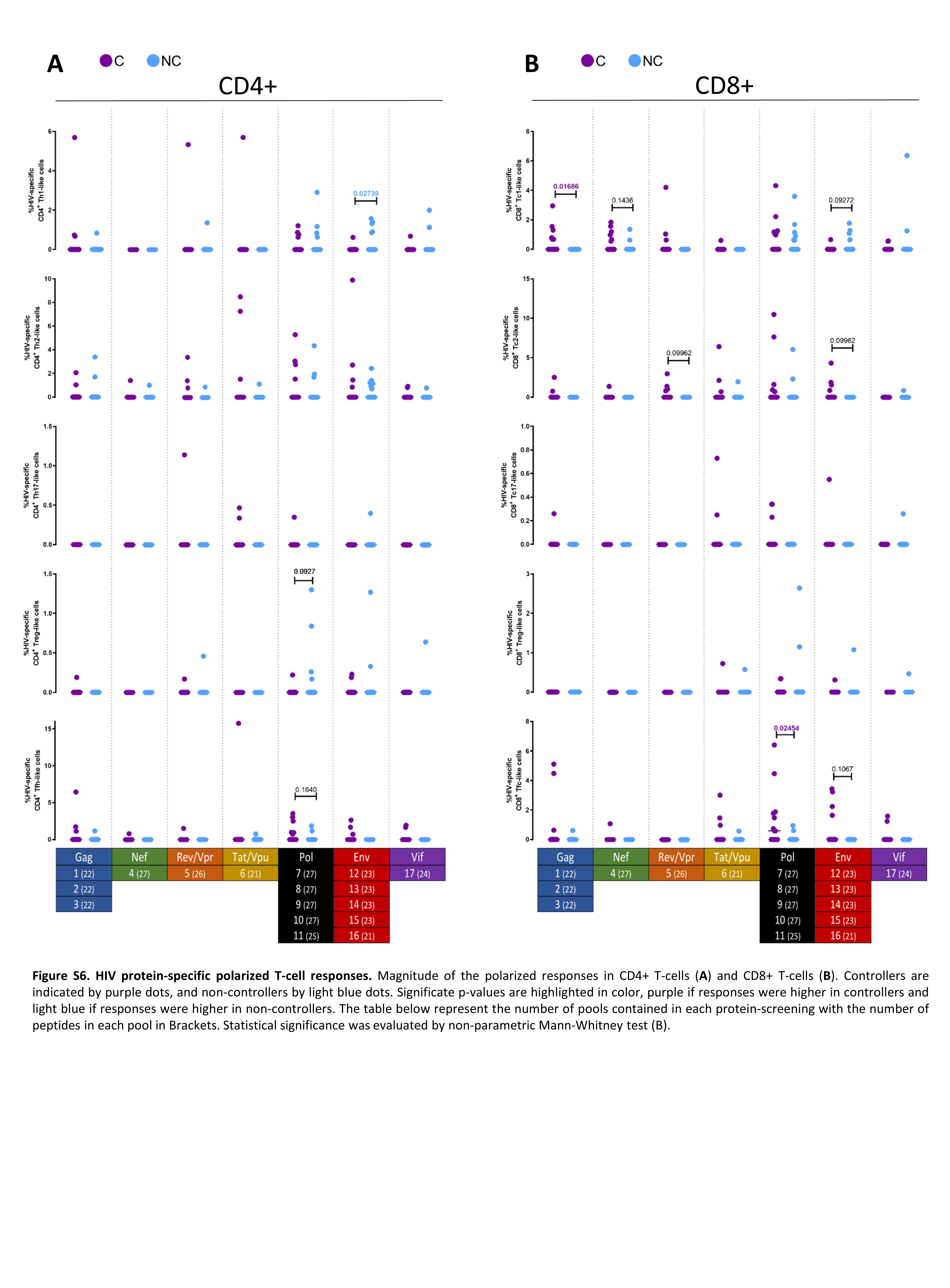

Supplement: Supplementary file 7 [file Image_6.tif]

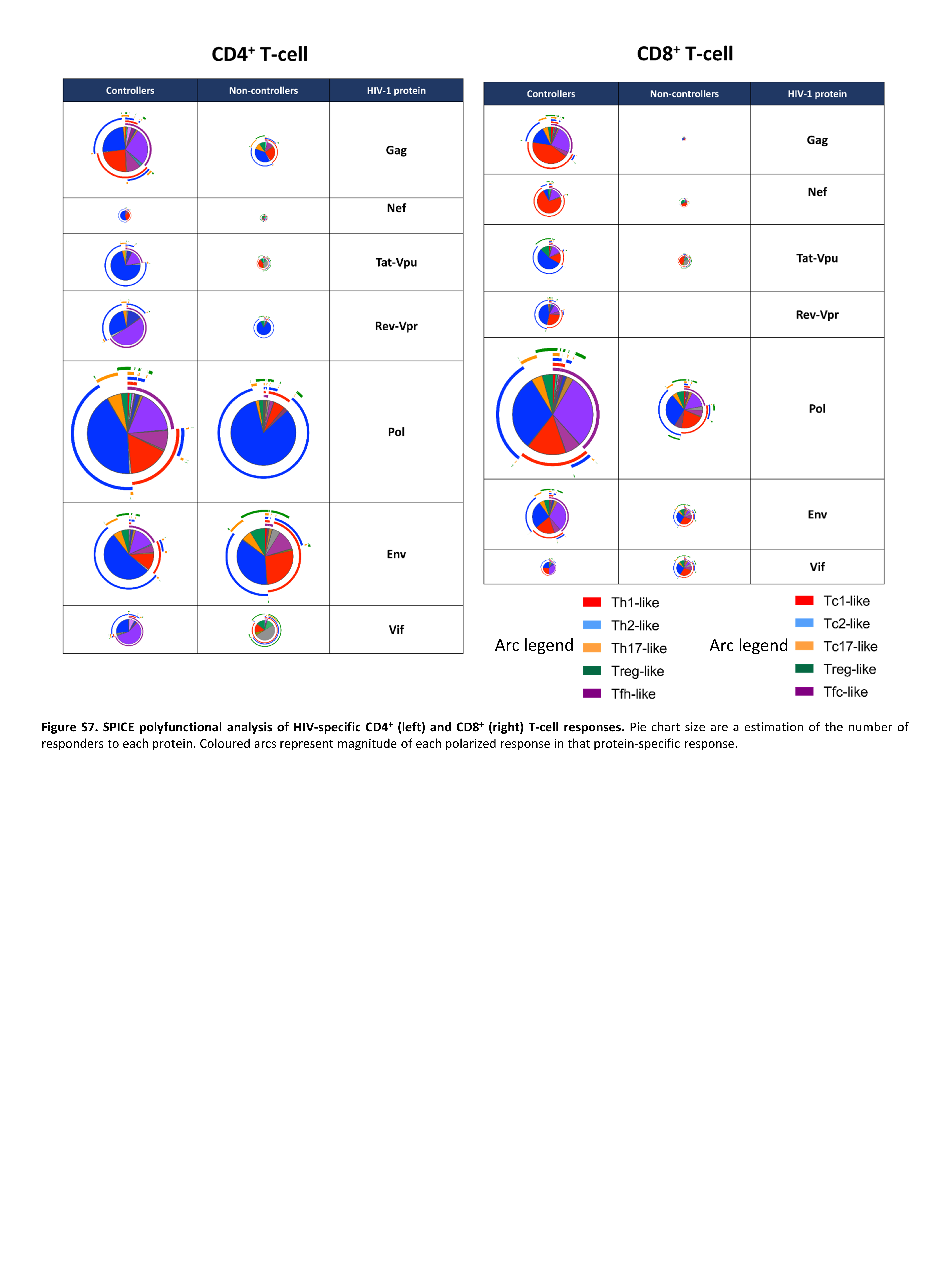

Supplement: Supplementary file 8 [file Image_7.tif]

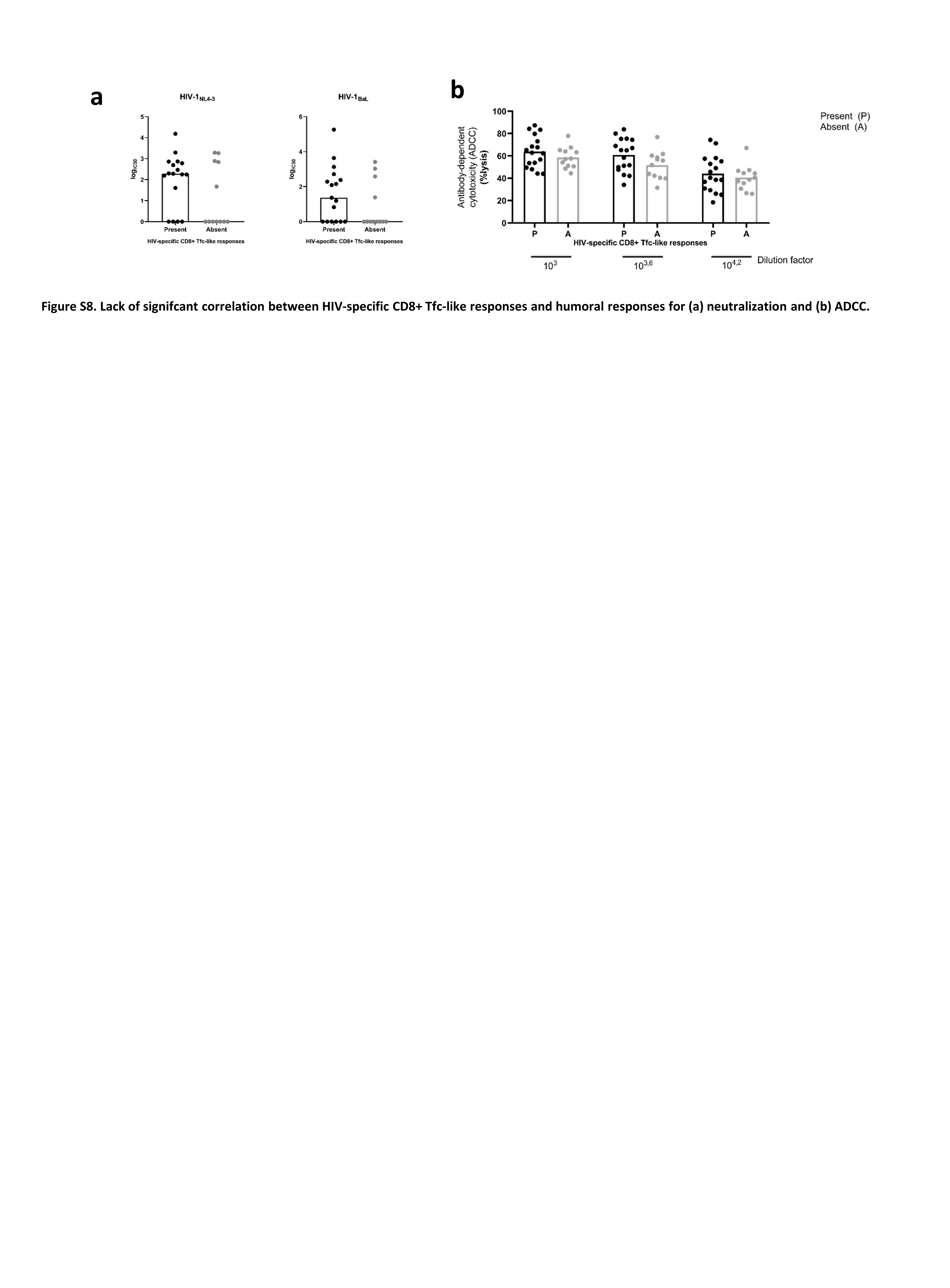

Supplement: Supplementary file 9 [file Image_8.tif]
